# Competing chaperone pathways in α‐synuclein disaggregation and aggregation dynamics

**DOI:** 10.1002/pro.70296

**Published:** 2025-09-13

**Authors:** Nicola K. Auld, Shannon McMahon, Nicholas R. Marzano, Antoine M. van Oijen, Heath Ecroyd

**Affiliations:** ^1^ Molecular Horizons and School of Science University of Wollongong Wollongong New South Wales Australia; ^2^ Faculty of Medicine and Health University of Sydney Sydney New South Wales Australia

**Keywords:** disaggregation, Hsp27, Hsp70 system, molecular chaperones, αB‐crystallin, α‐Synuclein

## Abstract

The aggregation of the protein α‐synuclein into amyloid fibrils and their subsequent deposition into large proteinaceous inclusions is a pathological hallmark of several neurodegenerative diseases, including Parkinson's disease. Molecular chaperones, including the small heat‐shock proteins (sHsps) and the Hsp70 chaperone system, are known to interact with α‐synuclein fibrils, preventing further aggregation and disaggregating fibrillar species, respectively. However, it remains unclear if sHsps co‐operate with the Hsp70 chaperones to potentially improve the kinetics or effectiveness of Hsp70‐mediated disaggregation and how disaggregation kinetics are influenced by aggregation‐prone α‐synuclein monomers. Using thioflavin‐T assays, we demonstrate that the sHsps Hsp27 (HSPB1) and αB‐crystallin (HSPB5) do not synergize with the Hsp70 chaperones during α‐synuclein seed fibril disaggregation. Moreover, the addition of monomeric α‐synuclein with fibril seeds results in increased aggregation that overwhelms Hsp70‐mediated disaggregation. Overall, these results suggest that while Hsp70 and sHsp chaperones are independently capable of binding to and inhibiting fibril elongation, they do not have synergistic effects on disaggregation. Furthermore, Hsp70‐mediated disaggregation is ineffectual in the presence of physiological concentrations of α‐synuclein monomers, conditions that actually lead to further α‐synuclein aggregation. Overall, these data may offer insight into factors that lead to the failure of the Hsp70 chaperones to clear cells of α‐synuclein aggregates that lead to neurodegenerative disease.

## 1 INTRODUCTION

Many neurodegenerative diseases are associated with the formation and accumulation of stable and highly ordered filamentous protein aggregates known as amyloid fibrils, the deposition of which into insoluble intracellular or extracellular protein inclusions serves as pathological hallmarks of neurodegenerative diseases (Aguzzi & O'Connor, 2010; Chiti & Dobson, 2017). More specifically, the aggregation and deposition of the protein α‐synuclein into Lewy body inclusions in the brain are characteristic features of Parkinson's disease (PD), multiple system atrophy (MSA) and dementia with Lewy bodies (Spillantini et al., 1997), collectively known as synucleinopathies. The formation of α‐synuclein fibrils from monomers occurs through a nucleation‐dependent polymerization process (Wood et al., 1999). During the initial lag phase, monomeric α‐synuclein molecules associate with each other to form oligomers or pre‐fibrillar species, with a characteristic cross‐β‐sheet structure (Sunde et al., 1997). These nuclei or “seeds” form the basis for elongation into fibrils, which proceeds rapidly through the addition of monomers to the ends of these nuclei until the system reaches an equilibrium. Secondary nucleation events, including fibril breakage or fragmentation and nucleation of monomers on the surface of existing fibrils, can accelerate the aggregation process (Buell et al., 2014; Cohen et al., 2013; Gaspar et al., 2017; Knowles et al., 2009). While mature fibrils, seeds, and early oligomers of α‐synuclein are cytotoxic, the widely accepted consensus is that oligomeric and seed forms of α‐synuclein are more cytotoxic than mature fibrils (reviewed in Alam et al., 2019; Cascella et al., 2022).

It is well recognized that molecular chaperones are a critical first line of defense against protein aggregation (Chiti & Dobson, 2017; Hartl et al., 2011). However, more recent work has demonstrated that some molecular chaperones are also involved in the disassembly of aggregates, including amyloid fibrils (Glover & Lindquist, 1998; Nillegoda et al., 2015; Rampelt et al., 2012; Shorter, 2011; Wentink et al., 2020). In particular, purified components of the Hsp70 chaperone system, which consists of an Hsp70 chaperone together with an Hsp40 co‐chaperone and a nucleotide exchange factor (NEF), are capable of ATP‐dependent disaggregation of fibrils formed by α‐synuclein (Beton et al., 2022; Franco, Gracia, et al., 2021; Gao et al., 2015; Schneider et al., 2021; Wentink et al., 2020), tau (Nachman et al., 2020) and huntingtin (Scior et al., 2018). A model of Hsp70‐mediated disaggregation of α‐synuclein fibrils suggests that the binding of DNAJB1 (an Hsp40) to the fibril promotes the recruitment and clustering of HspA8 (a constitutively expressed Hsp70; Hsc70) at the ends of fibrils (Beton et al., 2022), a process facilitated by the NEF Hsp110 (also referred to as HspH2, HspA4 and Apg‐2). The association of this high molecular mass chaperone complex generates an entropic pulling force that allows the disassembly of α‐synuclein fibrils (Beton et al., 2022, Franco, Gracia, et al., 2021, Schneider et al., 2021, Wentink et al., 2020). There remains conjecture as to how this process occurs, with some suggesting a rapid “unzipping” of protofilaments generating monomeric products (Beton et al., 2022, Franco, Gracia, et al., 2021, Schneider et al., 2021), while others report that potentially toxic oligomers and small fragments may also be generated (Beton et al., 2022; Gao et al., 2015; Jäger et al., 2024; Tittelmeier et al., 2020). These studies have led to the proposal that the Hsp70 machinery is a potential therapeutic target for neurodegenerative diseases. However, in the context of disease, Hsp70‐mediated disaggregation would occur in cells in the presence of free monomeric α‐synuclein, which is estimated to constitute up to 1% of cytosolic neuronal protein (Iwai et al., 1995). It is therefore important to assess the efficiency of chaperone‐mediated disaggregation of α‐synuclein fibrils in the presence of free monomeric α‐synuclein, as free monomers in solution can potentially enhance the kinetics of aggregation. In addition, it remains to be explored whether other molecular chaperones impact the disaggregation activity of the Hsp70 chaperone system.

The small heat‐shock proteins (sHsps) are a class of molecular chaperones known to inhibit the fibrillar aggregation of α‐synuclein through interactions with both monomeric and oligomeric species (Bruinsma et al., 2011; Cox et al., 2014; Rekas et al., 2004; Waudby et al., 2010). Moreover, sHsps can form stable complexes with mature α‐synuclein fibrils (Cox et al., 2016; Selig et al., 2020; Waudby et al., 2010). There are reports that the binding of sHsps to amyloid fibrils can lead to disaggregation; for example, the sHsp αB‐crystallin (HSPB5) dissociates oligomeric forms of the amyloid fibril forming protein β_2_ microglobulin into monomers (Esposito et al., 2013; Stepanenko et al., 2020) and both Hsp27 (HSPB1) and αB‐crystallin are capable of disaggregating amyloid fibrils of apolipoprotein C (Binger et al., 2013; Selig et al., 2020). A previous study demonstrated that neither αB‐crystallin nor Hsp27 alone was found to be capable of disaggregating α‐synuclein fibrils (Selig et al., 2020). However, Duennwald et al. (2012) reported that αB‐crystallin enhanced the disaggregation of α‐synuclein fibrils by the Hsp70 chaperone system, albeit this work involved an (atypical) 10‐day incubation of fibrils with chaperones to observe disaggregation, which is a much slower rate of disaggregation than typically observed for the Hsp70 chaperone system alone (Franco, Gracia, et al., 2021; Schneider et al., 2021; Wentink et al., 2020). Hence, the capacity for sHsps to enhance disaggregation by the Hsp70 chaperone system remains unclear and warrants further investigation.

Here, we investigate whether the sHsps αB‐crystallin or Hsp27 impacts the Hsp70‐mediated disaggregation of fibrillar forms of α‐synuclein. We find no evidence for these sHsps having a synergistic role in α‐synuclein fibril disaggregation by the Hsp70 chaperone system. Instead, these two chaperone classes potentially compete for binding sites at fibril ends. Furthermore, we find that, at physiologically relevant concentrations, the presence of α‐synuclein monomers overwhelms the disaggregation capacity of the Hsp70 chaperones, resulting in an overall increase in fibril formation.

## 2 RESULTS

### 2.1 The Hsp70 chaperone system removes α‐synuclein monomers from fibril ends

**FIGURE 1:**
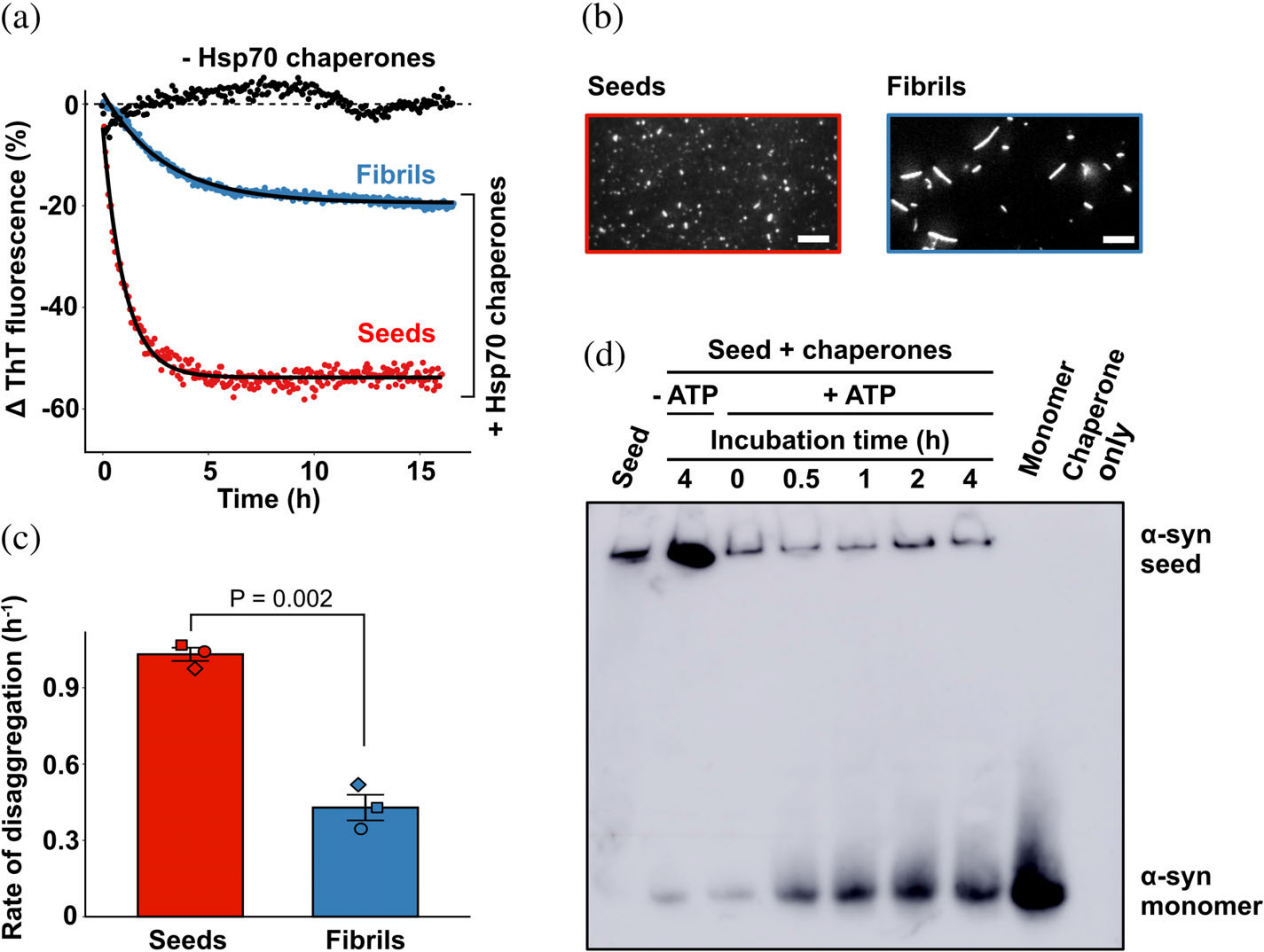
Disaggregation of α‐synuclein seeds and fibrils by the Hsp70 chaperone system results in the release of free α‐synuclein monomers. α‐Synuclein seeds or fibrils (2 μM) were incubated in the presence or absence of the Hsp70 chaperones (2 μM HspA8, 1 μM DNAJB1 and 0.2 μM Hsp110) at 30°C for up to 8 h. (a) Kinetic traces of α‐synuclein seed and fibril disaggregation as monitored by the change in Thioflavin‐T (ThT) fluorescence over time. Data is representative of three independent experiments and fitted to a one‐phase exponential decay model. (b) Representative total internal reflection fluorescence (TIRF) microscopy image of α‐synuclein seeds and fibrils incubated in 50 mM phosphate‐6‐hydroxy‐2,5,7,8‐tetramethyl chroman‐2‐carboxylic acid buffer supplemented with 5 μM α‐cyanostilbene derivative dye. Scale bars represent 5 μm. (c) The rate of seed and fibril disaggregation as determined from one‐phase exponential decay model. Data are means (± SEM) of three independent experiments (*n* = 3) and were analyzed using a student's *t*‐test. (d) Aliquots from the disaggregation reaction of α‐synuclein seeds were taken at the timepoints indicated and added to 50 mM EDTA to quench the reaction. Samples were subject to native‐polyacrylamide gel electrophoresis (PAGE) and immunoblotting with an anti‐α‐synuclein antibody. A sample of monomeric α‐synuclein (5 μM) was also included. The blot shown is representative of three independent experiments.

Hsp70‐mediated disaggregation of fibrillar forms of α‐synuclein is hypothesized to progress rapidly from fibril ends generating monomeric products (Franco, Gracia, et al., 2021) but may also include fibril fragmentation (Beton et al., 2022; Gao et al., 2015; Tittelmeier et al., 2020). To investigate this, recombinant α‐synuclein was aggregated to form mature fibrils, which were subsequently sonicated to produce small amyloid fragments (seeds). As expected, seeds and mature fibrils of α‐synuclein were successfully disaggregated by the Hsp70 system chaperone machinery (Figure 1a) in an ATP‐dependent manner (Figure S1); however, the rate and amount of disaggregation were significantly greater for the α‐synuclein seeds (Figure 1c, *t*_2.99_ = 10.61, *p* = 0.00182), in agreement with previous results (Franco, Gracia, et al., 2021). TIRF microscopy images (Figure 1b) confirmed that seed fragments were shorter than mature fibrils (Figure S2) and represented an increase in the number of fibril ends.

To investigate the products of Hsp70‐mediated disaggregation, aliquots taken during the disaggregation reaction were quenched with the addition of 50 mM ethylenediaminetetraacetic acid (EDTA) to chelate MgCl_2_ and stop ATP hydrolysis by HspA8 and analyzed by native‐polyacrylamide gel electrophoresis (PAGE) electrophoresis and subsequent immunoblotting (Figure 1d). Incubation of α‐synuclein seeds with the Hsp70 chaperone system and ATP resulted in a decrease in intensity of the higher molecular weight species, indicative of a decrease in the amount of seed. Correspondingly, there was an increase in intensity of a low molecular weight band corresponding to monomeric α‐synuclein. Of note, no other oligomeric forms of α‐synuclein (e.g., dimers or other multimers) were detected during the disaggregation process. Thus, these data indicate that the Hsp70‐mediated disaggregation of α‐synuclein seeds primarily occurs through the liberation of monomeric units from fibril ends.

### 2.2 Hsp70‐mediated disaggregation is outcompeted by aggregation‐prone monomeric α‐synuclein

**FIGURE 2:**
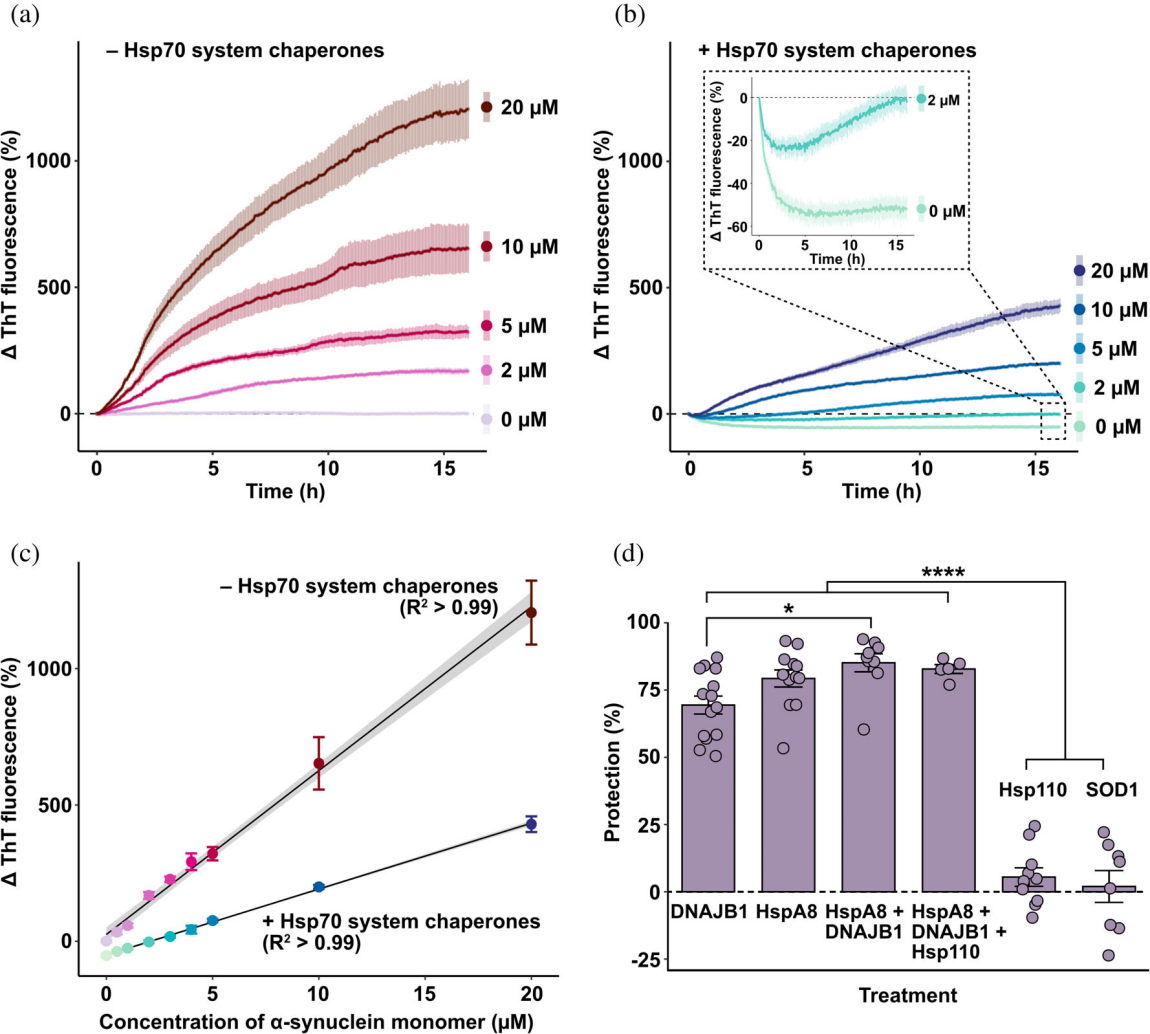
The presence of free monomeric α‐synuclein reduces the capacity of the Hsp70 chaperone system to mediate disaggregation and shifts the reaction toward fibril aggregation. α‐synuclein seeds (2 μM) were incubated with increasing concentrations of monomeric α‐synuclein (0–20 μM) in the (a) absence or (b) presence of the Hsp70 chaperones (2 μM HspA8, 1 μM DNAJB1 and 0.2 μM Hsp110) in disaggregation buffer containing 20 μM Thioflavin‐T (ThT) at 30°C. The change in ThT fluorescence was monitored over time. Data are means (± SEM) of three independent experiments (*n* = 3). Dashed lines indicate position of no change in ThT fluorescence. Inset panel in (b) shows a zoomed‐in view of the 0 and 2 μM monomeric α‐synuclein traces. (c) The endpoint normalized ThT fluorescence for each treatment as a function of the concentration of free monomeric α‐synuclein present. Data were fit to a linear regression model. (d) α‐synuclein seeds (2 μM) were incubated in the absence of nucleotide with 10 μM monomeric α‐synuclein and individual components of the Hsp70 chaperone system (or an equivalent amount of the non‐chaperone control protein superoxide dismutase 1 [SOD1]) in disaggregation buffer containing 20 μM ThT at 30°C. The percentage protection against seed elongation afforded by each chaperone combination is shown. Data are means (± SEM) of at least five independent experiments (*n* ≥ 5) and analyzed using a one‐way analysis of variance with Tukey's honestly significant difference post hoc testing where * and **** indicate *p* < 0.05 and *p* ≤ 0.0001, respectively (comparisons not indicated were not significant; *p* > 0.05).

Given that disaggregation of α‐synuclein fibrillar species in the cellular environment occurs in the presence of monomeric α‐synuclein, we investigated the capacity of the Hsp70 chaperones to disaggregate α‐synuclein seed fibrils when additional free monomeric α‐synuclein was present. As expected, there was a concentration‐dependent increase in the overall change in Thioflavin‐T (ThT) fluorescence associated with fibril elongation in the presence of higher concentrations of monomeric α‐synuclein (Figure 2a). Interestingly, only a relatively modest concentration of α‐synuclein monomers (2 μM) negated the decrease in ThT fluorescence associated with Hsp70‐mediated disaggregation of α‐synuclein seed fibrils, shifting the overall kinetics toward aggregation, as evidenced through a net increase in ThT fluorescence (Figure 2b). When the Hsp70 chaperones were present, the overall level of fibril aggregation stimulated by monomeric α‐synuclein was reduced by up to approximately three‐fold (Figure 2c). Equivalent protection against seed elongation was also seen with the addition of an ATP regeneration system (Figure S3), implying that this behavior is not a consequence of ATP depletion or adenosine diphosphate enrichment. Furthermore, the constitutively expressed HspA8 and its co‐chaperone DNAJB1 were responsible for the inhibition of fibril elongation, offering significantly more protection against seed elongation than the NEF Hsp110 and the non‐chaperone control protein superoxide dismutase 1 (SOD1) (Figure 2d, *F*_5,52_ = 98.99, *p* < 0.0001). Together, these data suggest that in the presence of free monomeric α‐synuclein, the Hsp70 chaperone system can suppress the amount of fibrillar aggregation that would otherwise occur but is unable to effectively promote fibril disaggregation.

### 2.3 sHsps do not promote Hsp70‐mediated disaggregation of α‐synuclein fibrils

**FIGURE 3:**
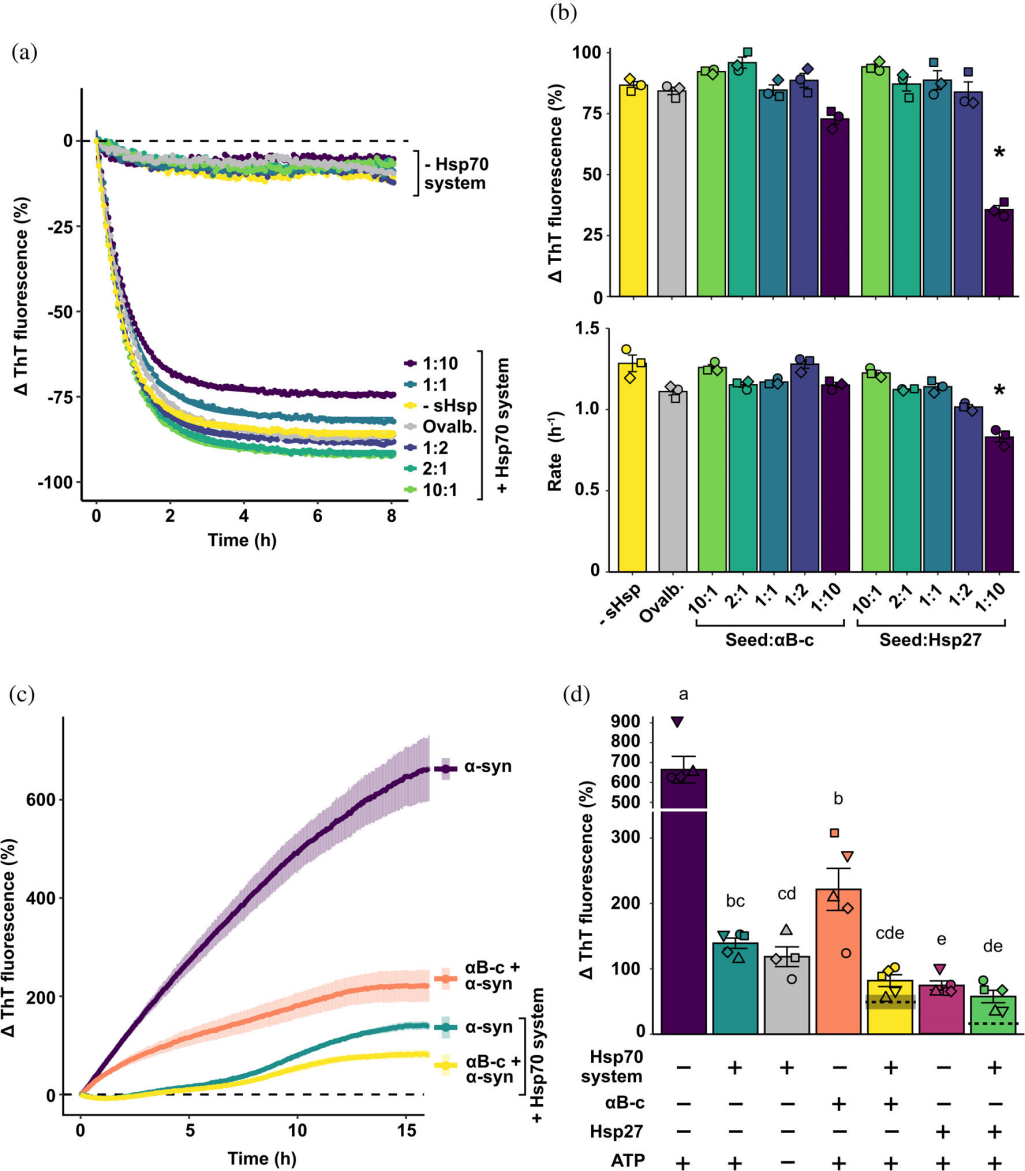
Small heat‐shock proteins (sHsps) do not co‐operate with Hsp70 system chaperones during disaggregation but act independently to prevent α‐synuclein seed fibril elongation. (a) A representative trace of the α‐synuclein seeds pre‐incubated with sHsps, with and without the Hsp70 chaperone system. Data are from technical duplicates of each sample, and those with the Hsp70 chaperones were fitted to a one‐phase exponential decay model from which the (b) rate and amount of disaggregation were determined. Statistically significant differences between the means of all sHsp concentrations, ovalbumin, and the seed‐only control were assessed using a one‐way analysis of variance (ANOVA) and Tukey's honestly significant difference (HSD) post hoc test for the rate of disaggregation (*p* < 0.0001) and the overall amount of disaggregation (*p* < 0.0001). Treatments that were significantly different from both the seed‐only (‐sHsp) and non‐chaperone (ovalbumin) controls are indicated by *. (c) Representative traces showing α‐synuclein seeds incubated with combinations of sHsps, the Hsp70 system and ATP in addition to 10 μM monomeric α‐synuclein in disaggregation buffer containing 20 μM ThT at 30°C for 16 h. Data are means (± SEM) of at least 4 independent experiments. (d) The overall normalized change in ThT fluorescence (as a percentage) was analyzed via a one‐way ANOVA (*p* < 0.0001) followed by a Tukey's HSD post hoc test. Different lower‐case letters above bars indicate significant differences between groups. Expected additive effects of the combination of each sHsp with the Hsp70 chaperones are indicated as dashed lines (mean ± SEM, with standard error of the mean (SEM) denoted by shading: In some cases the SEM is too small to be visualized).

Given that sHsps are known to bind to α‐synuclein fibrils (Cox et al., 2016; Waudby et al., 2010) and have some capacity to disaggregate fibrils formed by apolipoprotein C‐II (Selig et al., 2020) an β_2_ microglobulin (Esposito et al., 2013), we first examined whether the presence of sHsps alone resulted in a decrease in the ThT fluorescence associated with α‐synuclein seed fibrils. No decrease in ThT fluorescence was observed when α‐synuclein seeds were incubated with sHsps alone at any of the concentrations tested (Figure 3a). This result is in agreement with previous data that indicated that neither αB‐crystallin nor Hsp27 were capable of causing appreciable levels of disaggregation of fibrillar forms of α‐synuclein in isolation (Selig et al., 2020).

Next, we explored whether sHsps influence the Hsp70‐mediated disaggregation of α‐synuclein seeds. To facilitate sHsp binding, the seeds were pre‐incubated with either αB‐crystallin or Hsp27, at varying molar ratios (from 10:1–1:10; α‐synuclein seed:sHsp). Upon dilution of sHsp‐bound seeds into buffer containing the Hsp70 system chaperones, the characteristic exponential decay in ThT fluorescence was observed, indicative of disaggregation (Figure 3a). Analysis by one‐way analysis of variance (ANOVA) (Figure 3b, *F*_11,24_ = 43.2, *p* < 0.0001) and subsequent post hoc testing demonstrated that at low molar ratios (10:1, 2:1, 1:1, and 1:2 seed:sHsp) there was no significant difference in the amount of disaggregation that occurred in the presence or absence of the sHsps (*p* ≥ 0.0828). At the highest molar ratio tested (1:10 seed:sHsp), the addition of Hsp27 resulted in significantly lower levels of seed disaggregation compared to when the sHsp was not present or when the non‐chaperone control protein ovalbumin was present (*p* < 0.0001). With regard to the rate at which disaggregation occurs, again only the highest concentration of Hsp27 (a molar ratio of 1:10 seed:Hsp27) significantly decreased the rate of disaggregation compared to both the absence of the sHsp or when ovalbumin was present (*p* < 0.0001). This suggests that high concentrations of Hsp27 relative to the amount of α‐synuclein seed decreases both the rate of Hsp70‐mediated disaggregation and the amount of disaggregation that occurs.

Since sHsps are potent suppressors of α‐synuclein fibrillar aggregation (Bruinsma et al., 2011; Cox et al., 2016), we next investigated whether these sHsps further reduce the net elongation of α‐synuclein seeds observed in samples containing seeds, the Hsp70 chaperone system, and free monomeric α‐synuclein. While the combination of αB‐crystallin and the Hsp70 chaperones significantly reduced the elongation of fibrils compared to when αB‐crystallin was present alone (*F*_6,27_ = 46.6, *p* < 0.0001), there was no significant difference compared to when the Hsp70 chaperones were present alone (Figure 3c,d). Similar results were obtained when Hsp27 was present in place of αB‐crystallin. The expected change in ThT fluorescence arising from the addition of each sHsp with the Hsp70 chaperones, in the presence of both seeds and free monomer, was calculated (indicated by dashed lines in Figure 3d). As neither Hsp27 nor αB‐crystallin in combination with the Hsp70 chaperones achieved the level of aggregation suppression that would be indicative of synergy, these data indicate that, under these conditions, the sHsps and Hsp70 chaperones act independently to inhibit the elongation of α‐synuclein seeds by monomeric α‐synuclein.

### 2.4 Hsp70‐mediated disaggregation promotes α‐synuclein aggregation in the presence of sHsps at physiologically relevant concentrations

**FIGURE 4:**
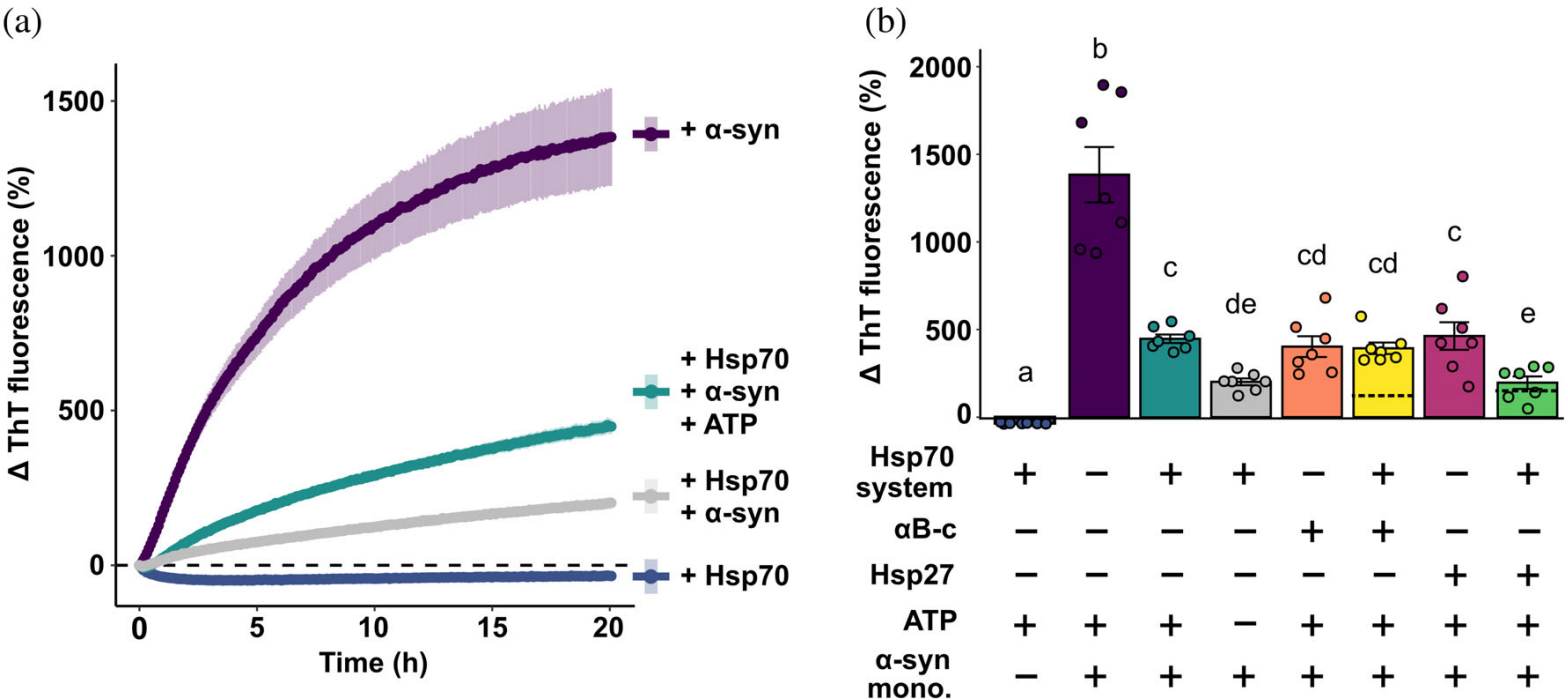
The disaggregation of α‐synuclein seeds at physiological concentrations of Hsp70 and sHsps. Physiologically relevant concentrations of Hsp70 (14 μM) and αB‐crystallin or Hsp27 (20 μM) were incubated with α‐synuclein seeds (2 μM) and free monomeric α‐synuclein (50 μM). Select traces are shown in (a) where data are means (± SEM) of seven independent experiments. The overall normalized change in Thioflavin‐T (ThT) fluorescence (as a percentage) (b) was analyzed via a one‐way analysis of variance (*p* < 0.0001) with a Tukey's honestly significant difference post hoc test. Different lower‐case letters above bars indicate significant differences between groups. Expected additive effects of the combination of each sHsp with the Hsp70 chaperones are indicated as dashed lines (mean ± SEM, with SEM denoted by shading: In some cases the SEM is too small to be visualized).

Given the interplay between monomeric and fibrillar forms of α‐synuclein when chaperones are present, we sought to examine the impact of physiologically relevant concentrations of free monomeric α‐synuclein and the molecular chaperones with regard to the aggregated state of α‐synuclein. To do so, we used concentrations of α‐synuclein and the chaperones based on previously reported values: namely 50 μM monomeric α‐synuclein (Iwai et al., 1995; Wilhelm et al., 2014); 20 μM sHsps (Mymrikov et al., 2020); and 14 μM HspA8 (Moran Luengo et al., 2018) with concentrations of DNAJB1 and Hsp110 so as to maintain the 1:0.5:0.1 (HspA8:DNAJB1:Hsp110) molar ratio used previously for the Hsp70 chaperone system. Of note, when seed fibrils were incubated with physiologically relevant concentrations of free monomeric α‐synuclein, there were significant increases in ThT fluorescence (*F*_7,48_ = 62.4, *p* < 0.0001) even when the Hsp70 chaperone disaggregation machinery was present (Figure 4). Strikingly, the presence of ATP with the Hsp70 chaperones led to a significant increase in ThT fluorescence compared to when ATP was absent (Figure 4b, *p* = 0.0105), suggesting that active Hsp70‐mediated disaggregation (facilitated by ATP) enhances α‐synuclein aggregation under these conditions. In line with previous findings (see Figure 3c,d), no evidence of synergy between chaperone classes was observed. Specifically, the combination of αB‐crystallin and the Hsp70 chaperones displayed some evidence of antagonism and the combination of Hsp27 and the Hsp70 chaperones was observed to be independent or simply additive (Figure 4b).

## 3 DISCUSSION

The chaperone network plays a key role in regulating the aggregation of α‐synuclein, a process that, when left unchecked, leads to the formation of amyloid fibrils, which are pathological hallmarks of the synucleinopathies. Of therapeutic interest are chaperones that interact with fibrillar forms of α‐synuclein, potentially leading to their disaggregation. The ATP‐dependent Hsp70 system chaperones constitute a disaggregation machinery that is capable of disaggregating α‐synuclein fibrils (Gao et al., 2015; Wentink et al., 2020) whereas the ATP‐independent sHsps bind directly to α‐synuclein fibrils, preventing their further elongation, but are not independently capable of mediating disaggregation (Selig et al., 2020). However, it remained to be established whether the binding of sHsps to α‐synuclein fibrils impacts Hsp70‐mediated amyloid fibril disaggregation. Here, we provide evidence that these two functionally distinct classes of molecular chaperones do not have synergistic effects when it comes to mediating α‐synuclein fibril disaggregation. Furthermore, we demonstrate that the presence of free monomeric α‐synuclein significantly reduces the efficacy of Hsp70 chaperones to disassemble α‐synuclein fibrils in vitro.

The overwhelming majority of studies investigating chaperone‐mediated α‐synuclein fibril disaggregation involve the study of exclusively fibrillar forms of α‐synuclein. However, in a physiological setting within the cell, α‐synuclein aggregates form and elongate within a pool of monomeric α‐synuclein. Mouse models of synucleinopathies reveal a prion‐like spreading and amplification of fibrillar α‐synuclein following inoculation of mice with pre‐formed fibrils (Ayers et al., 2018; Luk et al., 2012; Masuda‐Suzukake et al., 2013), highlighting the role that monomeric α‐synuclein plays in mediating the spread of disease‐related pathology. Hence, we sought to investigate the kinetics of Hsp70‐mediated α‐synuclein seed disaggregation in the presence of free monomeric α‐synuclein. Our data show that, in samples containing Hsp70 chaperones and fibrillar forms of α‐synuclein, as the amount of free monomeric α‐synuclein increases, the ThT fluorescence kinetics are characterized by an increasingly brief disaggregation phase followed by elongation of the fibrils (see Figure 2b). Moreover, the addition of a relatively low (2 μM) amount of free monomeric α‐synuclein was sufficient to negate the overall reduction in ThT fluorescence associated with the disaggregation facilitated by the Hsp70 chaperones. The decrease in disaggregation observed in the presence of α‐synuclein monomers may be due to competition between monomeric and aggregated forms of α‐synuclein for Hsp70 chaperone binding, whereby increased levels of monomers in solution act to sequester the Hsp70 chaperones from binding to fibrils. However, Hsp70 and DNAJ isoforms related to those used in this study have been demonstrated to preferentially bind to fibrillar, as opposed to monomeric, forms of α‐synuclein (Aprile et al., 2017; Hinault et al., 2010). Alternatively, the kinetic and thermodynamic advantages of the association of monomers with fibrils may surpass those of chaperone‐mediated disaggregation, resulting in the predominance of seed elongation over disaggregation. Regardless, the relatively modest concentration of α‐synuclein required to negate the effects of Hsp70 chaperone‐mediated disaggregation suggests that a delicate balance exists between the competing forces of seed elongation and disaggregation in the cellular environment.

While disaggregation was limited in the presence of free α‐synuclein monomers, the presence of Hsp70 chaperones under these conditions did suppress the increase in ThT fluorescence associated with seed elongation (see Figure 2c). This observation suggests that, under these circumstances, the Hsp70 and DNAJ chaperones can bind α‐synuclein to prevent further aggregation, in agreement with previous results (Klucken et al., 2004; Luk et al., 2008; McLean et al., 2002; Pemberton et al., 2011). Together, these results highlight the ability of the Hsp70 chaperone system to not only act as a “disaggregase” but additionally in a “holdase” role to mitigate α‐synuclein seed elongation in the presence of α‐synuclein monomers. Furthermore, the addition of sHsps to the system containing Hsp70 chaperones, monomeric α‐synuclein, and seeds, resulted in no synergistic suppression of seed elongation (Figure 3c,d). As both sHsps (Selig et al., 2022; Waudby et al., 2010) and the Hsp70 chaperones (Beton et al., 2022; Franco, Gracia, et al., 2021; Schneider et al., 2021) are thought to bind preferentially to fibril ends, the lack of synergy observed may be due to competition for binding to these end regions of the fibrils.

We note incomplete or partial disaggregation of α‐synuclein fibrils by the Hsp70 chaperone system, evidenced by the presence of fibrillar α‐synuclein species at the end of the 4 h incubation period, by which time the decrease in ThT fluorescence has reached a plateau (Figure 1a,d). Similar findings have been reported in both biochemical (Gao et al., 2015) and atomic force microscopy (Beton et al., 2022) studies indicating that some fibrillar species remain unaffected by the disaggregation machinery (Beton et al., 2022). As α‐synuclein is able to form a variety of polymorphic oligomeric and fibrillar structures (reviewed in Mehra et al., 2021; Yoo et al., 2022), with some morphologies suggested to be more amenable to chaperone‐mediated disaggregation than others (Fricke et al., 2024; Jäger et al., 2024), it is possible that structural differences may account for the persistence of some fibrillar species seen in this work.

It has been demonstrated previously that truncation of α‐synuclein, resulting in the removal of negative charges at the C‐terminal region (Franco, Cuellar, et al., 2021) and the removal of key N‐terminal HspA8 interaction sites at residues 1–10 and 37–43 (Redeker et al., 2012; Wentink et al., 2020), can reduce or completely abolish disaggregation by the Hsp70 system chaperones. These results highlight the need for HspA8 and its co‐chaperone DNAJB1 to access specific regions of α‐synuclein in order for productive binding and disaggregation of fibrils to proceed. It follows that fibrils that are not fully disaggregated may persist, proliferate, and amplify—a mechanism that may lead to the accumulation of certain α‐synuclein polymorphs in deposits such as Lewy bodies. Furthermore, incomplete disaggregation may lead to the generation of α‐synuclein species that are more capable of prion‐like spreading in a disease context (Bett et al., 2017; Silveira et al., 2005).

Whereas the co‐aggregation of sHsps with amorphously aggregating client proteins is crucial for efficient resolubilization and disassembly of these aggregates by the Hsp70 system chaperones (Gonçalves et al., 2021; Nillegoda et al., 2015; Rampelt et al., 2012), only a single study has previously investigated the effect of sHsps on Hsp70‐mediated amyloid disaggregation (Duennwald et al., 2012). While this previous work reported that αB‐crystallin potentiated the Hsp70‐mediated disaggregation of α‐synuclein fibrils, the rate of disaggregation reported was atypically slow (up to 10 days incubation was required to observe ~50% disaggregation) and, over these extended timeframes, αB‐crystallin alone liberated α‐synuclein into the supernatant. In contrast, and in agreement with previous observations (Selig et al., 2020), we found that over more typical timeframes for the study of the in vitro disaggregation of α‐synuclein fibrils (typically 1–5 h for the half‐time of the disaggregation reaction) (Beton et al., 2022; Franco, Gracia, et al., 2021; Schneider et al., 2021; Wentink et al., 2020), sHsps alone were not capable of disaggregating α‐synuclein seeds to any appreciable extent. This suggests that the binding of sHsps to α‐synuclein fibrils acts to stabilize the fibril, preventing further elongation and decreasing toxicity (Cox et al., 2018; Selig et al., 2020). Moreover, when sHsp‐bound α‐synuclein fibrils were exposed to the Hsp70 disaggregation machinery, we found no evidence to suggest a synergistic effect of these two chaperone systems on α‐synuclein disaggregation. Instead, we observed that when free α‐synuclein monomer is present, the two chaperone classes are independent with regard to suppressing further α‐synuclein aggregation (Figure 3d). Of note, the Bliss independence model we have used to test for synergistic effects does assume complete mechanistic independence of the two effectors, that is, that the mechanism(s) through which the Hsp70 and sHsp chaperones interact with α‐synuclein fibrils are independent of each other. Further research is required to confirm whether this is the case. Nevertheless, it is clear from our data that the two chaperone classes do not act synergistically with regard to the rate of disaggregation. Instead, under conditions in which the relative concentration of the chaperones is high, there may be competition between sHsps and the Hsp70 chaperones for binding to the ends of α‐synuclein fibrils, since this is where monomers are removed during disaggregation.

Increasing the concentrations of sHsps, Hsp70 chaperones, and monomeric α‐synuclein present with α‐synuclein seeds to approximate physiologically relevant levels led to an overall increase in ThT fluorescence over time. Thus, under conditions where the presence of ATP allows for efficient Hsp70 binding and disaggregation of fibrils, disaggregation activity is overwhelmed by the rate at which seeds elongate and aggregate in the presence of monomers. The observation that the presence of ATP promotes seed aggregation (Figure 4b) suggests that Hsp70 disaggregation activity may facilitate the proliferation and spread of seeding‐competent species in a disease context. These results support previous findings showing that α‐synuclein (Jäger et al., 2024) and tau (Nachman et al., 2020) fibrils subjected to Hsp70‐mediated disaggregation had a greater seeding capacity than fibrils not subjected to Hsp70‐mediated disaggregation. In addition, depletion of Hsp110 in a *Caenorhabditis elegans* model of disaggregation reduced both Hsp70‐mediated disaggregation as well as aggregated α‐synuclein foci and toxicity (Tittelmeier et al., 2020). Together, these data suggest that while disaggregation may reduce the length of α‐synuclein fibrils and eliminate some aggregated species, it may ultimately lead to increased α‐synuclein aggregation.

## 4 CONCLUSIONS

The disaggregation of α‐synuclein fibrils by the Hsp70 chaperones is a complex and dynamic process. The results of this work indicate that although Hsp70 chaperones can efficiently reduce aggregate levels in isolation, the addition of monomeric α‐synuclein reduces or negates this effect, with Hsp70 chaperones acting in “holdase” roles to prevent fibril elongation. Additionally, our results suggest that rather than synergizing with Hsp70 chaperones during disaggregation, sHsps potentially compete with Hsp70 chaperones for productive binding sites on the ends of α‐synuclein fibrils. Furthermore, at physiologically relevant concentrations, Hsp70 disaggregation activity may lead to an increase in the number of aggregates, potentially leading to a proliferation of species that can amplify synucleinopathy disease pathology.

## 5 MATERIALS AND METHODS

### 5.1 Protein expression, extraction, and purification of α‐synuclein, Hsp27, and αB‐crystallin

A single colony of BL21(DE3) *Escherichia coli* (*E. coli*) cells transformed with a plasmid encoding either α‐synuclein, Hsp27, or αB‐crystallin was used to inoculate a starter culture (~100 mL) of lysogeny broth (LB; 5% [w/v] yeast, 10% [w/v] NaCl, 10% [w/v] tryptone, pH 7.4) supplemented with appropriate antibiotics (100 μg/mL ampicillin or 50 μg/mL kanamycin). Starter cultures were grown overnight at 37°C with constant agitation at 180 rpm. The starter culture was then diluted 20‐fold into LB media containing the appropriate antibiotic to ensure maintenance of the plasmid. Cultures were incubated at 37°C with shaking at 180 rpm until an optical density at 600 nm (OD_600_) of ~0.8 was reached. The expression of α‐synuclein, Hsp27, or αB‐crystallin was induced by the addition of 0.25 mM isopropyl‐β‐D‐1‐thiogalactopyranoside (IPTG) and cultures were incubated at 37°C for 4 h. The cells were then harvested by centrifugation at 5000 × *g* for 10 min at 4°C, and the pellet was stored at −20°C until the recombinant protein was extracted.

In order to purify recombinant Hsp27 and αB‐crystallin, cell pellets were thawed and resuspended in ice‐cold lysis buffer (50 mM Tris, 100 mM NaCl and 1 mM EDTA, 0.5 mM phenylmethanesulfonyl fluoride (PMSF), pH 8.0) containing a cOmplete™ EDTA‐free protease inhibitor cocktail (Sigma‐Aldrich, St. Louis, MO, USA), lysozyme (0.5 mg/mL) and DNase I (3.0 μg/mL). Resuspended cells were incubated for 30 min at 4°C with gentle rocking and then probe sonicated using the Sonifer® 250 Digital cell disruptor (Branson Ultrasonics Corporation, Brookfield, CT, USA) for 3 min at 45% amplitude (10 s on/20 s off). The lysates were clarified by at least two rounds of centrifugation at 24,000 × *g* for 30 min at 4°C prior to being filtered through a 0.45 μm filter. The bacterial lysates were loaded onto a HiPrep™ DEAE FF 16/10 column (GE HealthCare, Chicago, IL, USA) pre‐equilibrated in 20 mM Tris, 1 mM EDTA, 3 mM sodium azide, 0.5 mM PMSF (pH 8.5) and proteins were eluted at 2.5 mL/min over eight column volumes using a linear gradient (0–200 mM NaCl). Fractions (5 mL) identified to contain Hsp27 or αB‐crystallin were pooled and concentrated to 5 mL using an Amicon® stirred filtration cell (Merck Millipore, Burlington, MA, USA) before being loaded onto a HiPrep™ 26/60 Sephacryl® S‐300 HR column (Cytiva, Marlborough, MA, USA) equilibrated in 50 mM phosphate buffer (PB) with 0.5 mM PMSF (pH 7.4). Proteins were eluted at 1.5 mL/min into 10 mL fractions prior to being pooled, concentrated using a 10 K MWCO Pierce™ Protein Concentrator (Thermo Fisher Scientific, Waltham, MA, USA) and stored at −80°C until required.

Purification of recombinant α‐synuclein was performed as described previously (Buell et al., 2014) with modifications. Briefly, bacterial cells were resuspended in lysis buffer (100 mM Tris and 10 mM EDTA, pH 8.0) supplemented with protease inhibitor, prior to being subjected to three slow freeze–thaw cycles (−20°C to room temperature with gentle rocking). The lysate was then probe sonicated as described above before being heated to 90°C for 20 min to remove bacterial host proteins. The lysate was centrifuged at 22,000 × *g* for 20 min at 4°C. Streptomycin sulfate was added to the cleared lysate at a concentration of 10 mg/mL and was stirred at 4°C for 20 min. This process of centrifugation and subsequent stirring in the presence of streptomycin sulfate was repeated once before the lysate was clarified by a final round of centrifugation at 24,000 × *g* for 20 min at 4°C. The lysate was filtered through a 0.45 μm filter and applied to a HiPrep™ DEAE FF 16/10 column (Cytiva) equilibrated in 20 mM Tris, 1 mM EDTA, 3 mM sodium azide (pH 8.5). The protein was eluted at 2.5 mL/min over four column volumes using a linear gradient (0–500 mM NaCl) and 5 mL fractions were collected. Fractions identified to contain α‐synuclein were pooled and loaded onto a HiLoad® 16/600 Superdex® 75 pg size‐exclusion column (GE HealthCare) pre‐equilibrated in 50 mM PB (pH 7.5) and the protein was eluted into 4 mL fractions at a flow rate of 1.0 mL/min. Fractions containing purified, recombinant α‐synuclein were pooled and the protein was concentrated using a 10 K MWCO Pierce™ Protein Concentrator prior to storage at −80°C.

### 5.2 Protein expression, extraction, and purification of HspA8, DNAJB1, and Hsp110

Plasmids encoding recombinant 6 × His‐SUMO‐HspA8 (also known as Hsc70), 6 × His‐SUMO‐DNAJB1 (also known as Hdj‐1) or 6 × His‐SUMO‐Hsp110 (also known as HspA4, HspH2 and Apg‐2) were transformed into Rosetta™ (DE3) *E. coli*, and single colonies were used to inoculate ~100 mL 2× yeast extract tryptone media (YT; 10% [w/v] yeast extract, 5% [w/v] NaCl, 16% [w/v] tryptone, pH 7.0) media supplemented with kanamycin (50 μg/mL) and chloramphenicol (25 μg/mL). The starter cultures were incubated overnight at 37°C with shaking at 180 rpm before being diluted 20‐fold into fresh 2 × YT media supplemented with kanamycin (50 μg/mL) only. Once cultures reached an OD_600_ of between 0.6 and 0.8, expression was induced using 1 mM IPTG. Cells were grown for a further 4 (HspA8 and DNAJB1) to 6 h (Hsp110) at 30°C with constant shaking before being harvested by centrifugation at 5000 × *g* for 10 min at 4°C. Cell pellets were stored at −20°C until extraction. Cells containing recombinant HspA8, DNAJB1, or Hsp110 were resuspended in lysis buffer (50 mM HEPES‐KOH (pH 7.4), 5 mM MgCl_2_, 2 mM β‐mercaptoethanol (BME), 20 mM imidazole, 10% glycerol) supplemented with a protease inhibitor cocktail, DNase I (3.0 μg/mL), lysozyme (0.5 mg/mL) and either 750 mM (DNAJB1) or 300 mM (HspA8 and Hsp110) KCl. Cells were sonicated as described previously and centrifuged (24,000 × *g*, 30 min, 4°C) twice before being passed through a 0.45 μm filter.

HspA8, DNAJB1, and Hsp110 were purified by loading each bacterial cell lysate (~50 mL) at 1 mL/min onto a 5 mL HisTrap™ HP Sepharose column (Cytiva) equilibrated with the appropriate lysis buffer. The column was then washed at 5 mL/min with a low salt buffer (50 mM HEPES‐KOH (pH 7.4), 5 mM MgCl_2_, 2 mM BME, 20 mM imidazole, 10% glycerol) containing either 500 mM (DNAJB1) or 100 mM (HspA8 and Hsp110) KCl until the absorbance at 280 nm had returned to baseline. For HspA8 and Hsp110, an additional low salt buffer containing 10 mM ATP was applied to the column and the flow was stopped for 30 min at room temperature. The column was washed again with low salt buffer until the absorbance at 280 nm had stabilized. Recombinant proteins were eluted at 1 mL/min from the column into 5 mL fractions using the appropriate low salt buffer containing 300 mM imidazole. To cleave the N‐terminal 6 × His‐SUMO tag, those fractions containing the protein of interest were pooled and dialyzed overnight at 4°C into a cleavage buffer (50 mM HEPES‐KOH (pH 7.4), 5 mM MgCl_2_, 2 mM BME, 20 mM imidazole, 100 mM KCl, 10% glycerol) in the presence of the SUMO protease, 6 × His‐tagged ULP1 (4 μg/mL per mg of recombinant protein). Cleaved proteins were loaded back onto the HisTrap™ HP Sepharose column under the same conditions as described previously to separate the 6 × His‐SUMO tag. The proteins were then loaded onto a HiLoad® 16/600 Superdex® 200 size‐exclusion column (Cytiva) pre‐equilibrated in 50 mM HEPES‐KOH (pH 7.4), 5 mM MgCl_2_, 2 mM BME, 10% glycerol, and 500 mM (DNAJB1) or 100 mM (HspA8 and Hsp110) KCl. Proteins were eluted from the column at 1.5 mL/min into 10 mL fractions. Fractions containing the protein of interest were pooled and concentrated using a 10 K MWCO Pierce™ Protein Concentrator and stored at −80°C until use.

### 5.3 Preparation of α‐synuclein seeds and mature fibrils

To form α‐synuclein amyloid seeds, 100 μM monomeric α‐synuclein was incubated in 50 mM PB (pH 6.3) in low protein binding tubes (Thermo Fisher Scientific) for 24 h at 45°C with constant stirring using a Spinbar® magnetic stirring flea (Sigma‐Aldrich) on a Magnetic Stirrer & Hot Plate combination with ‘Thermostat’ temperature control (Industrial Equipment and Control, Thornbury, VIC, Australia). Samples were probe sonicated (Digital Sonifier 250, Branson Ultrasonics Corporation) at 30% power for 30 s (10 s on/30 s off) and were then incubated as before for a further 24 h. The sample was centrifuged (20,000 × *g*, 40 min, 4°C) to separate fibrils (which form a pellet) from soluble monomeric protein. A wash step was performed whereby the pellet was gently resuspended in 50 mM PB (pH 7.4) and centrifuged again (20,000 × *g*, 15 min, 4°C). Once the soluble fraction was removed and discarded, the pellet was resuspended in 50 mM PB (pH 7.4). The fibrils were sonicated a final time, as before, to fragment them into seeds. The final concentration of α‐synuclein in these samples, reported as monomeric equivalent of α‐synuclein, was determined via a bicinchoninic acid assay and aliquots were frozen in liquid nitrogen and stored at −80°C.

Seeds of α‐synuclein (2.5 μM of monomeric equivalent) were elongated into mature fibrils by the addition of 100 μM of monomeric α‐synuclein in 50 mM PB (pH 7.4) and incubation for 48 h at 45°C without shaking. Following the incubation period, samples were centrifuged (20,000 × *g*, 40 min, 4°C) to separate the mature fibrils from monomeric and smaller oligomeric forms of α‐synuclein. A wash step was performed whereby the pellet was gently resuspended in 50 mM PB (pH 7.4) and centrifuged again (20,000 × *g*, 15 min, 4°C). Once the soluble fraction was removed and discarded, the pellet containing the mature fibrils was resuspended in 50 mM PB (pH 7.4) and stored at 4°C.

### 5.4 Assay to monitor the disaggregation of fibrillar forms of α‐synuclein by molecular chaperones

To monitor the kinetics of the disaggregation of α‐synuclein seeds, 2 μM α‐synuclein seeds (monomeric equivalent) were incubated with 2 μM HspA8, 1 μM DNAJB1, and 0.2 μM Hsp110 in disaggregation buffer consisting of 50 mM HEPES‐KOH, 50 mM KCl, 5 mM MgCl_2_, 2 mM dithiothreitol, 0.05% (v/v) Tween‐20 (pH 7.5) supplemented with 20 μM ThT. Samples were prepared in duplicate and incubated in a clear‐bottom 384‐well microplate (Greiner Bio‐One, Kremsmünster, Austria) at 30°C in a FLUOstar Optima plate reader (BMG LABTECH, Melbourne, VIC, Australia) for 15 min for temperature equilibration of the ThT. Following this, pre‐heated ATP (or Milli‐Q® water as a control) was added to all samples (5 mM final ATP concentration) and the plate was shaken for 30 s. The subsequent disaggregation of α‐synuclein fibrils was monitored by measuring the fluorescence of ThT using excitation/emission filters of 440/490 nm, respectively, for a maximum of 16 h.

To investigate the effect of sHsps on the capacity of the Hsp70 chaperone system to disaggregate α‐synuclein seeds, the seeds were first pre‐incubated with sHsps (αB‐crystallin or Hsp27) or ovalbumin (as a non‐chaperone control protein) at molar ratios of 10:1, 2:1, 1:1, 1:2, or 1:10 (α‐synuclein:sHsp/ovalbumin) for 30 min at room temperature to facilitate binding (Cox et al., 2018). Following this incubation, samples were diluted into disaggregation buffer containing 2 μM HspA8, 1 μM DNAJB1, 0.2 μM Hsp110, and 20 μM ThT in a clear‐bottom 384‐well black microplate (Greiner Bio‐One) and incubated for 15 min prior to the addition of pre‐heated 5 mM ATP. The ThT fluorescence of these samples was monitored as described above. For disaggregation experiments performed in the presence of monomeric α‐synuclein, 10 μM monomeric α‐synuclein was added to the disaggregation buffer along with the Hsp70 chaperone system.

For disaggregation experiments performed at physiological concentrations of α‐synuclein and molecular chaperones, the following concentrations were used: 50 μM for monomeric α‐synuclein (Iwai et al., 1995; Wilhelm et al., 2014); 20 μM for the sHsps (Mymrikov et al., 2020); 14 μM for HspA8 (Moran Luengo et al., 2018) with DNAJB1 and Hsp110 concentrations of 7 μM and 1.4 μM, respectively, to maintain the 1:0.5:0.1 molar ratio of HspA8:DNAJB1:Hsp110 used in previous experiments. For some experiments where indicated, reactions were supplemented with an ATP regeneration system (8 mM phosphoenolpyruvic acid and 20 ng/μL pyruvate kinase (Sigma‐Aldrich, P7768)).

### 5.5 Assay to monitor the elongation of α‐synuclein seeds in the presence of molecular chaperones

Samples of α‐synuclein seeds (2 μM of monomeric equivalent) were incubated with up to 20 μM monomeric α‐synuclein, 2 μM sHsp (or SOD1 as a non‐chaperone control protein), 2 μM HspA8, 1 μM DNAJB1, and 0.2 μM Hsp110. As described previously, samples were prepared in duplicate in disaggregation buffer supplemented with 20 μM ThT, and ThT fluorescence was measured for up to 20 h at 30°C using a FLUOstar Optima plate reader (BMG LABTECH).

### 5.6 Native‐PAGE and immunoblotting

To monitor the disaggregation of α‐synuclein seeds by the Hsp70 chaperone system via Native‐PAGE, 8 μM α‐synuclein seeds (monomeric equivalent) were incubated with 8 μM HspA8, 4 μM DNAJB1, and 0.8 μM Hsp110 and 5 mM ATP in disaggregation buffer in low protein binding tubes (Thermo Fisher Scientific) and incubated at 30°C for a maximum of 4 h. Aliquots (50 μL) were taken from this mixture with 50 mM EDTA added (to a final volume of 55 μL) to quench the reaction for 20 min at room temperature before being placed on ice. Samples containing α‐synuclein seeds only, monomeric α‐synuclein only, or α‐synuclein seeds with chaperones in the absence of ATP were included as controls. Samples were subsequently diluted into Native‐PAGE loading buffer (final concentrations: 0.81M Tris–HCl [pH 6.8], 16% [v/v] glycerol, 0.01% bromophenol blue) before being loaded into the wells of a hand‐cast 6% (w/v) polyacrylamide gel (4.7 mL Milli‐Q® water, 3.7 mL Tris [1M, pH 8.8], 1.5 mL 40% [w/v] bis‐acrylamide [37.5:1], 25 μL 10% [w/v] ammonium persulfate, 25 μL tetramethylethylenediamine). Samples were subjected to electrophoresis using a Mini‐Protean® Tetra Cell system (Bio‐Rad, Hercules, CA, USA) for 2.5 h at 150 V in ice‐cold Native‐PAGE running buffer (0.25M Tris, 1.92M glycine, pH 8.8).

Following Native‐PAGE, samples were transferred to a polyvinylidene difluoride membrane at 100 V in ice‐cold transfer buffer (25 mM Tris base, 192 mM glycine, 20% [v/v] methanol, pH 8.3). The membrane was blocked overnight at 4°C in Tris‐buffered saline (TBS; 50 mM Tris base, 150 mM NaCl, pH 7.6) containing 5% (w/v) skim milk powder. Membranes were then probed with an anti‐α‐synuclein antibody (mouse monoclonal, Abcam, Cambridge, UK, catalog number ab1903) diluted 1:2000 in TBS supplemented with 0.05% (v/v) Tween‐20 (TBS‐T) containing 5% (w/v) skim milk powder. Primary antibody incubation was performed for 2 h at room temperature with gentle rocking. The membrane was washed four times (each for 10 min) in TBS‐T before being incubated with the secondary antibody (anti‐mouse IgG horseradish peroxidase‐conjugated antibody; rabbit polyclonal, Sigma‐Aldrich; #A9044) diluted 1:5000 in TBS‐T containing 5% (w/v) skim milk powder for 1 h with gentle rocking at room temperature. Finally, the membrane was washed as before in TBS‐T before visualization of proteins of interest with SuperSignal® West Dura Extended Duration Chemiluminescent Substrate (Thermo Fisher Scientific) using a ChemiDoc™ Imaging System (Bio‐Rad).

### 5.7 ThT fluorescence data analysis

For each sample, background ThT fluorescence of buffer and chaperones was subtracted. At each time point, the fluorescence intensity of samples was calculated relative to the fluorescence intensity of samples containing α‐synuclein fibrils or seeds alone. Hence, the normalized change in ThT fluorescence reflects the ThT fluorescence intensity at any given time point as a percentage of the fibril/seed‐only control. Data from the technical duplicates were averaged and, where relevant, each sample was fit to a one‐phase exponential decay model in R (R Core Team, 2022). For each sample, the rate and endpoint of the fitted model were extracted to quantify the rate of disaggregation and disaggregation yield, respectively.

At the end of each experiment involving addition of monomeric α‐synuclein, the ability of each chaperone mixture to inhibit α‐synuclein aggregation was calculated using the following formula: protection (%) = 100 × (Δ*F*_N_ – Δ*F*_C_)/Δ*F*_N_ where Δ*F*_C_ and Δ*F*_N_ represent the normalized overall change in ThT fluorescence in the presence and absence of chaperones, respectively.

Where appropriate, to test for synergism or antagonism, the fractional effect of each chaperone class (sHsp or Hsp70 system) in reducing α‐synuclein seed aggregation was calculated using:

(1) $$E_{\text{chap}}=\frac{\left({\Delta F}_{N}–{\Delta F}_{C}\right)}{{\Delta F}_{N}},$$

where Δ*F*_C_ and Δ*F*_N_ represent the normalized overall change in ThT fluorescence in the presence and absence of chaperones, respectively. Then, the expected additive effect (*E*_sHsp+Hsp70_) of combining these two chaperone classes was calculated in accordance with the Bliss independence model (Bliss, 1939) using:

(2) $$\;E_{\text{sHsp}+\mathrm{Hsp}70}=E_{\text{sHsp}}+E_{\mathrm{Hsp}70}-\left(E_{\text{sHsp}}\times E_{\mathrm{Hsp}70}\right),$$

where *E*_sHsp_ and *E*_Hsp70_ refer to the fractional effect of either sHsp or the Hsp70 chaperone system, and *E*_sHsp+Hsp70_ refers to the expected additive effect of the combination. If the measured fractional effect was greater than the *E*_sHsp+Hsp70_, the combination of chaperones was considered synergistic, and if the measured fractional effect was less than *E*_sHsp+Hsp70_ the combination of chaperones was considered antagonistic.

### 5.8 TIRF microscopy

Samples were imaged using a custom‐built TIRF microscopy setup as previously detailed (Marzano et al., 2022). To prepare coverslips for imaging, 24 mm by 24 mm glass coverslips were cleaned by alternate cycles of water bath sonication (Digital benchtop ultrasonic cleaner 250HD, Soniclean, Dudley Park, SA, Australia) for 20 min in 100% ethanol and KOH (5 M), repeated three times. A final sonication in Milli‐Q® water for 15 min was performed. Coverslips were dried with compressed nitrogen gas and incubated with poly‐L‐lysine solution (0.01% [w/v]) for 30 min to immobilize α‐synuclein fibrils to the surface. Following this incubation, coverslips were rinsed with Milli‐Q® water, dried using compressed nitrogen gas, and stored in a custom humidity chamber before use. Coverslips were coupled to the objective lens by immersion oil before loading of the sample.

All α‐synuclein fibrils and seeds were diluted approximately 1:500 in 50 mM PB (pH 7.4) containing 6 mM 6‐hydroxy‐2,5,7,8‐tetramethyl chroman‐2‐carboxylic acid (TROLOX) and an α‐cyanostilbene derivative (ASCP) dye (5 μM) (Marzano et al., 2020). Samples (50 μL) were deposited directly onto the surface of the coverslip and illuminated using a circularly polarized laser (Sapphire 488–150 CW, Coherent, Saxonburg, PA, USA) at 200 mW cm^−2^ at 488 nm. The camera (Andor iXon Life 897 EMCCD, Oxford Instruments, Abingdon, UK) was operated at −70°C in frame transfer mode at 20 Hz with an electron multiplication gain of 700 and a pixel distance of 160 nm (in sample space). Imaging of the ASCP fluorescence was performed at 500 ms per image. Samples were at room temperature during imaging (approximately 20°C).

All TIRF images were initially corrected for electronic offset and uneven excitation beam distribution across the field of view. To determine the length of α‐synuclein seed and fibril contours, corrected images were analyzed using the “Ridge Detection” plugin (Steger, 1998) in Fiji ImageJ (Schindelin et al., 2012), providing the length of seed or fibril contours (in pixels). These lengths were converted to nm and deconvolved using the following formulae:

(3) $$\text{Deconvolved contour length}=\sqrt{{\text{(measured contour length)}}^{2}-{\text{(diffraction �limit)}}^{2},�}$$

where the diffraction limit is calculated as.

(4) $$\begin{array}{l} \text{Diffraction limit}=\frac{0.61\times \lambda }{\text{numerical aperture}}\\ \;=\frac{0.61\times 488\;\mathrm{nm}}{1.49}=199.8\;\mathrm{nm}. \end{array}$$

### 5.9 Statistics

All statistical analyses were performed in R (R Core Team, 2022), using ggbreak (Xu et al., 2021) (version 0.1.2), outliers (Komsta, 2022) (version 0.15), emmeans (Lenth, 2023) (version 1.8.9) and car (Fox & Weisberg, 2019) (version 3.1‐1) packages. Data were assessed for normality via the Shapiro–Wilk test and homoscedasticity via Cochran's *C* test. Where necessary, the overall change in ThT fluorescence (endpoint) data were log_10_ or square‐root transformed to improve normality and homoscedasticity. A one‐way ANOVA with Tukey's honestly significant difference (HSD) post hoc test was performed to determine statistically significant differences between groups in overall change in ThT fluorescence, rate of disaggregation, or overall amount of disaggregation. A P‐value of less than 0.05 was considered statistically significant. Where relevant, the results of Tukey's HSD post hoc testing were graphically summarized using a Compact Letter Display (Piepho, 2004), with different lower‐case letters above bars indicating significant differences between groups.

## AUTHOR CONTRIBUTIONS

**Nicola K. Auld:** Conceptualization; investigation; writing – original draft; methodology; formal analysis; data curation. **Shannon McMahon:** Writing – review and editing; resources. **Nicholas R. Marzano:** Writing – review and editing; supervision; methodology. **Antoine M. van Oijen:** Conceptualization; funding acquisition; writing – review and editing; supervision. **Heath Ecroyd:** Conceptualization; funding acquisition; writing – review and editing; project administration; supervision.

## CONFLICT OF INTEREST STATEMENT

None of the authors have any conflicts of interest to disclose.

## Supporting information


**Data S1.** Supporting Information.

## Data Availability

The data that support the findings of this study are available from the corresponding author upon reasonable request.

## References

[pro70296-bib-0001] Aguzzi A , O'Connor T . Protein aggregation diseases: pathogenicity and therapeutic perspectives. Nat Rev Drug Discov. 2010;9:237–248. 20190788 10.1038/nrd3050

[pro70296-bib-0002] Alam P , Bousset L , Melki R , Otzen DE . Α‐Synuclein oligomers and fibrils: a spectrum of species, a spectrum of toxicities. J Neurochem. 2019;150:522–534. 31254394 10.1111/jnc.14808

[pro70296-bib-0003] Aprile FA , Arosio P , Fusco G , Chen SW , Kumita JR , Dhulesia A , et al. Inhibition of α‐synuclein fibril elongation by Hsp70 is governed by a kinetic binding competition between α‐synuclein species. Biochemistry. 2017;56:1177–1180. 28230968 10.1021/acs.biochem.6b01178

[pro70296-bib-0004] Ayers JI , Riffe CJ , Sorrentino ZA , Diamond J , Fagerli E , Brooks M , et al. Localized induction of wild‐type and mutant alpha‐synuclein aggregation reveals propagation along neuroanatomical tracts. J Virol. 2018;92:e00586‐00518. 29976670 10.1128/JVI.00586-18 PMC6146706

[pro70296-bib-0005] Beton JG , Monistrol J , Wentink A , Johnston EC , Roberts AJ , Bukau BG , et al. Cooperative amyloid fibre binding and disassembly by the Hsp70 disaggregase. EMBO J. 2022;41:e110410. 35698800 10.15252/embj.2021110410 PMC9379549

[pro70296-bib-0006] Bett C , Lawrence J , Kurt TD , Orru C , Aguilar‐Calvo P , Kincaid AE , et al. Enhanced neuroinvasion by smaller, soluble prions. Acta Neuropathol Commun. 2017;5:32. 28431576 10.1186/s40478-017-0430-z PMC5399838

[pro70296-bib-0007] Binger KJ , Ecroyd H , Yang S , Carver JA , Howlett GJ , Griffin MD . Avoiding the oligomeric state: αB‐crystallin inhibits fragmentation and induces dissociation of apolipoprotein C‐II amyloid fibrils. FASEB J. 2013;27:1214–1222. 23159935 10.1096/fj.12-220657

[pro70296-bib-0008] Bliss CI . The toxicity of poisons applied jointly. Ann Appl Biol. 1939;26:585–615.

[pro70296-bib-0009] Bruinsma IB , Bruggink KA , Kinast K , Versleijen AA , Segers‐Nolten IM , Subramaniam V , et al. Inhibition of α‐synuclein aggregation by small heat shock proteins. Proteins. 2011;79:2956–2967. 21905118 10.1002/prot.23152

[pro70296-bib-0010] Buell AK , Galvagnion C , Gaspar R , Sparr E , Vendruscolo M , Knowles TP , et al. Solution conditions determine the relative importance of nucleation and growth processes in α‐synuclein aggregation. Proc Natl Acad Sci U S A. 2014;111:7671–7676. 24817693 10.1073/pnas.1315346111 PMC4040554

[pro70296-bib-0011] Cascella R , Bigi A , Cremades N , Cecchi C . Effects of oligomer toxicity, fibril toxicity and fibril spreading in synucleinopathies. Cell Mol Life Sci. 2022;79:174. 35244787 10.1007/s00018-022-04166-9 PMC8897347

[pro70296-bib-0012] Chiti F , Dobson CM . Protein misfolding, amyloid formation, and human disease: a summary of progress over the last decade. Annu Rev Biochem. 2017;86:27–68. 28498720 10.1146/annurev-biochem-061516-045115

[pro70296-bib-0013] Cohen SI , Linse S , Luheshi LM , Hellstrand E , White DA , Rajah L , et al. Proliferation of amyloid‐β42 aggregates occurs through a secondary nucleation mechanism. Proc Natl Acad Sci U S A. 2013;110:9758–9763. 23703910 10.1073/pnas.1218402110 PMC3683769

[pro70296-bib-0014] Cox D , Carver JA , Ecroyd H . Preventing α‐synuclein aggregation: the role of the small heat‐shock molecular chaperone proteins. Biochim Biophys Acta. 2014;1842:1830–1843. 24973551 10.1016/j.bbadis.2014.06.024

[pro70296-bib-0015] Cox D , Selig E , Griffin MD , Carver JA , Ecroyd H . Small heat‐shock proteins prevent α‐synuclein aggregation via transient interactions and their efficacy is affected by the rate of aggregation. J Biol Chem. 2016;291:22618–22629. 27587396 10.1074/jbc.M116.739250 PMC5077198

[pro70296-bib-0016] Cox D , Whiten DR , Brown JWP , Horrocks MH , San Gil R , Dobson CM , et al. The small heat shock protein Hsp27 binds α‐synuclein fibrils, preventing elongation and cytotoxicity. J Biol Chem. 2018;293:4486–4497. 29382725 10.1074/jbc.M117.813865 PMC5868268

[pro70296-bib-0017] Duennwald ML , Echeverria A , Shorter J . Small heat shock proteins potentiate amyloid dissolution by protein disaggregases from yeast and humans. PLoS Biol. 2012;10:e1001346. 22723742 10.1371/journal.pbio.1001346 PMC3378601

[pro70296-bib-0018] Esposito G , Garvey M , Alverdi V , Pettirossi F , Corazza A , Fogolari F , et al. Monitoring the interaction between β2‐microglobulin and the molecular chaperone αB‐crystallin by NMR and mass spectrometry: αB‐crystallin dissociates β2‐microglobulin oligomers. J Biol Chem. 2013;288:17844–17858. 23645685 10.1074/jbc.M112.448639 PMC3682583

[pro70296-bib-0019] Fox J , Weisberg S . An R companion to applied regression. California, USA: Sage Publications; 2019.

[pro70296-bib-0020] Franco A , Cuellar J , Fernandez‐Higuero JA , de la Arada I , Orozco N , Valpuesta JM , et al. Truncation‐driven lateral association of α‐synuclein hinders amyloid clearance by the Hsp70‐based disaggregase. Int J Mol Sci. 2021;22:e12983. 10.3390/ijms222312983 PMC8657883 34884786

[pro70296-bib-0021] Franco A , Gracia P , Colom A , Camino JD , Fernandez‐Higuero JA , Orozco N , et al. All‐or‐none amyloid disassembly via chaperone‐triggered fibril unzipping favors clearance of α‐synuclein toxic species. Proc Natl Acad Sci U S A. 2021;118:e2105548118. 34462355 10.1073/pnas.2105548118 PMC8433526

[pro70296-bib-0022] Fricke C , Kunka A , Norrild RK , Wang S‐Y , Dang TL , Wentink AS , et al. Thermodynamic stability modulates chaperone‐mediated disaggregation of α‐synuclein fibrils. bioRxiv [pre‐print]. 2024 10.1101/2024.12.19.629136 PMC12622358 41257205

[pro70296-bib-0023] Gao X , Carroni M , Nussbaum‐Krammer C , Mogk A , Nillegoda NB , Szlachcic A , et al. Human Hsp70 disaggregase reverses Parkinson's‐linked α‐synuclein amyloid fibrils. Mol Cell. 2015;59:781–793. 26300264 10.1016/j.molcel.2015.07.012 PMC5072489

[pro70296-bib-0024] Gaspar R , Meisl G , Buell AK , Young L , Kaminski CF , Knowles TPJ , et al. Secondary nucleation of monomers on fibril surface dominates α‐synuclein aggregation and provides autocatalytic amyloid amplification. Q Rev Biophys. 2017;50:e6. 29233218 10.1017/S0033583516000172

[pro70296-bib-0025] Glover JR , Lindquist S . Hsp104, Hsp70, and Hsp40: a novel chaperone system that rescues previously aggregated proteins. Cell. 1998;94:73–82. 9674429 10.1016/s0092-8674(00)81223-4

[pro70296-bib-0026] Gonçalves CC , Sharon I , Schmeing TM , Ramos CHI , Young JC . The chaperone HSPB1 prepares protein aggregates for resolubilization by HSP70. Sci Rep. 2021;11:17139. 34429462 10.1038/s41598-021-96518-x PMC8384840

[pro70296-bib-0027] Hartl FU , Bracher A , Hayer‐Hartl M . Molecular chaperones in protein folding and proteostasis. Nature. 2011;475:324–332. 21776078 10.1038/nature10317

[pro70296-bib-0028] Hinault MP , Cuendet AF , Mattoo RU , Mensi M , Dietler G , Lashuel HA , et al. Stable α‐synuclein oligomers strongly inhibit chaperone activity of the Hsp70 system by weak interactions with J‐domain co‐chaperones. J Biol Chem. 2010;285:38173–38182. 20847048 10.1074/jbc.M110.127753 PMC2992251

[pro70296-bib-0029] Iwai A , Masliah E , Yoshimoto M , Ge N , Flanagan L , Rohan de Silva H , et al. The precursor protein of non‐aβ component of Alzheimer's disease amyloid is a presynaptic protein of the central nervous system. Neuron. 1995;14:467–475. 7857654 10.1016/0896-6273(95)90302-x

[pro70296-bib-0030] Jäger S , Tittelmeier J , Dang TL , Bellande T , Redeker V , Buell AK , et al. Structural polymorphism of α‐synuclein fibrils alters pathway of Hsc70 mediated disaggregation. bioRxiv [pre‐print]. 2024 10.1101/2024.12.02.626355 PMC12623964 41053261

[pro70296-bib-0031] Klucken J , Shin Y , Masliah E , Hyman BT , McLean PJ . Hsp70 reduces α‐synuclein aggregation and toxicity. J Biol Chem. 2004;279:25497–25502. 15044495 10.1074/jbc.M400255200

[pro70296-bib-0032] Knowles TP , Waudby CA , Devlin GL , Cohen SI , Aguzzi A , Vendruscolo M , et al. An analytical solution to the kinetics of breakable filament assembly. Science. 2009;326:1533–1537. 20007899 10.1126/science.1178250

[pro70296-bib-0033] Komsta L . Tests for outliers. R package version 0.15. 2022. https://cran.r-project.org/web/packages/outliers/index.html. Accessed 17 Feb 2025.

[pro70296-bib-0034] Lenth R . emmeans: estimated marginal means, aka least‐squares means. R package version 1.8.9. 2023. https://CRAN.R-project.org/package=emmeans. Accessed 17 Feb 2025.

[pro70296-bib-0035] Luk KC , Kehm V , Carroll J , Zhang B , O'Brien P , Trojanowski JQ , et al. Pathological α‐synuclein transmission initiates Parkinson‐like neurodegeneration in nontransgenic mice. Science. 2012;338:949–953. 23161999 10.1126/science.1227157 PMC3552321

[pro70296-bib-0036] Luk KC , Mills IP , Trojanowski JQ , Lee VMY . Interactions between Hsp70 and the hydrophobic core of α‐synuclein inhibit fibril assembly. Biochemistry. 2008;47:12614–12625. 18975920 10.1021/bi801475r PMC2648307

[pro70296-bib-0037] Marzano NR , Paudel BP , van Oijen AM , Ecroyd H . Real‐time single‐molecule observation of chaperone‐assisted protein folding. Sci Adv. 2022;8:eadd0922. 36516244 10.1126/sciadv.add0922 PMC9750156

[pro70296-bib-0038] Marzano NR , Wray KM , Johnston CL , Paudel BP , Hong Y , van Oijen A , et al. An α‐cyanostilbene derivative for the enhanced detection and imaging of amyloid fibril aggregates. ACS Chem Nerosci. 2020;11:4191–4202. 10.1021/acschemneuro.0c00478 33226775

[pro70296-bib-0039] Masuda‐Suzukake M , Nonaka T , Hosokawa M , Oikawa T , Arai T , Akiyama H , et al. Prion‐like spreading of pathological α‐synuclein in brain. Brain. 2013;136:1128–1138. 23466394 10.1093/brain/awt037 PMC3613715

[pro70296-bib-0040] McLean PJ , Kawamata H , Shariff S , Hewett J , Sharma N , Ueda K , et al. TorsinA and heat shock proteins act as molecular chaperones: suppression of α‐synuclein aggregation. J Neurochem. 2002;83:846–854. 12421356 10.1046/j.1471-4159.2002.01190.x

[pro70296-bib-0041] Mehra S , Gadhe L , Bera R , Sawner AS , Maji SK . Structural and functional insights into α‐synuclein fibril polymorphism. Biomolecules. 2021;11:1419. 34680054 10.3390/biom11101419 PMC8533119

[pro70296-bib-0042] Moran Luengo T , Kityk R , Mayer MP , Rudiger SGD . Hsp90 breaks the deadlock of the Hsp70 chaperone system. Mol Cell. 2018;70:545–552.e549. 29706537 10.1016/j.molcel.2018.03.028

[pro70296-bib-0043] Mymrikov EV , Riedl M , Peters C , Weinkauf S , Haslbeck M , Buchner J . Regulation of small heat‐shock proteins by hetero‐oligomer formation. J Biol Chem. 2020;295:158–169. 31767683 10.1074/jbc.RA119.011143 PMC6952609

[pro70296-bib-0044] Nachman E , Wentink AS , Madiona K , Bousset L , Katsinelos T , Allinson K , et al. Disassembly of tau fibrils by the human Hsp70 disaggregation machinery generates small seeding‐competent species. J Biol Chem. 2020;295:9676–9690. 32467226 10.1074/jbc.RA120.013478 PMC7363153

[pro70296-bib-0045] Nillegoda NB , Kirstein J , Szlachcic A , Berynskyy M , Stank A , Stengel F , et al. Crucial HSP70 co‐chaperone complex unlocks metazoan protein disaggregation. Nature. 2015;524:247–251. 26245380 10.1038/nature14884 PMC4830470

[pro70296-bib-0046] Pemberton S , Madiona K , Pieri L , Kabani M , Bousset L , Melki R . Hsc70 protein interaction with soluble and fibrillar alpha‐synuclein. J Biol Chem. 2011;286:34690–34699. 21832061 10.1074/jbc.M111.261321 PMC3186418

[pro70296-bib-0047] Piepho H‐P . An algorithm for a letter‐based representation of all‐pairwise comparisons. J Comput Graph Stat. 2004;13:456–466.

[pro70296-bib-0048] R Core Team . R: a language and environment for statistical computing. Version 4.2.2. Vienna: R Foundation for Statistical Computing; 2022.

[pro70296-bib-0049] Rampelt H , Kirstein‐Miles J , Nillegoda NB , Chi K , Scholz SR , Morimoto RI , et al. Metazoan Hsp70 machines use Hsp110 to power protein disaggregation. EMBO J. 2012;31:4221–4235. 22990239 10.1038/emboj.2012.264 PMC3492728

[pro70296-bib-0050] Redeker V , Pemberton S , Bienvenut W , Bousset L , Melki R . Identification of protein interfaces between α‐synuclein, the principal component of Lewy bodies in Parkinson disease, and the molecular chaperones human Hsc70 and the yeast Ssa1p. J Biol Chem. 2012;287:32630–32639. 22843682 10.1074/jbc.M112.387530 PMC3463349

[pro70296-bib-0051] Rekas A , Adda CG , Andrew Aquilina J , Barnham KJ , Sunde M , Galatis D , et al. Interaction of the molecular chaperone αB‐crystallin with α‐synuclein: effects on amyloid fibril formation and chaperone activity. J Mol Biol. 2004;340:1167–1183. 15236975 10.1016/j.jmb.2004.05.054

[pro70296-bib-0052] Schindelin J , Arganda‐Carreras I , Frise E , Kaynig V , Longair M , Pietzsch T , et al. Fiji: an open‐source platform for biological‐image analysis. Nat Methods. 2012;9:676–682. 22743772 10.1038/nmeth.2019 PMC3855844

[pro70296-bib-0053] Schneider MM , Gautam S , Herling TW , Andrzejewska E , Krainer G , Miller AM , et al. The Hsc70 disaggregation machinery removes monomer units directly from α‐synuclein fibril ends. Nat Commun. 2021;12:5999. 34650037 10.1038/s41467-021-25966-w PMC8516981

[pro70296-bib-0054] Scior A , Buntru A , Arnsburg K , Ast A , Iburg M , Juenemann K , et al. Complete suppression of Htt fibrilization and disaggregation of Htt fibrils by a trimeric chaperone complex. EMBO J. 2018;37:282–299. 29212816 10.15252/embj.201797212 PMC5770855

[pro70296-bib-0055] Selig EE , Lynn RJ , Zlatic CO , Mok YF , Ecroyd H , Gooley PR , et al. The monomeric α‐crystallin domain of the small heat‐shock proteins αB‐crystallin and Hsp27 binds amyloid fibril ends. J Mol Biol. 2022;434:167711. 35777462 10.1016/j.jmb.2022.167711

[pro70296-bib-0056] Selig EE , Zlatic CO , Cox D , Mok YF , Gooley PR , Ecroyd H , et al. N‐ and C‐terminal regions of αB‐crystallin and Hsp27 mediate inhibition of amyloid nucleation, fibril binding, and fibril disaggregation. J Biol Chem. 2020;295:9838–9854. 32417755 10.1074/jbc.RA120.012748 PMC7380184

[pro70296-bib-0057] Shorter J . The mammalian disaggregase machinery: Hsp110 synergizes with Hsp70 and Hsp40 to catalyze protein disaggregation and reactivation in a cell‐free system. PLoS One. 2011;6:e26319. 22022600 10.1371/journal.pone.0026319 PMC3194798

[pro70296-bib-0058] Silveira JR , Raymond GJ , Hughson AG , Race RE , Sim VL , Hayes SF , et al. The most infectious prion protein particles. Nature. 2005;437:257–261. 16148934 10.1038/nature03989 PMC1513539

[pro70296-bib-0059] Spillantini MG , Schmidt ML , Lee VMY , Trojanowski JQ , Jakes R , Goedert M . α‐synuclein in Lewy bodies. Nature. 1997;388:839–840. 9278044 10.1038/42166

[pro70296-bib-0060] Steger C . An unbiased detector of curvilinear structures. IEEE Trans Pattern Anal Mach Intell. 1998;20:113–125.

[pro70296-bib-0061] Stepanenko OV , Sulatsky MI , Mikhailova EV , Stepanenko OV , Povarova OI , Kuznetsova IM , et al. Alpha‐B‐crystallin effect on mature amyloid fibrils: different degradation mechanisms and changes in cytotoxicity. Int J Mol Sci. 2020;21:7659. 33081200 10.3390/ijms21207659 PMC7589196

[pro70296-bib-0062] Sunde M , Serpell LC , Bartlam M , Fraser PE , Pepys MB , Blake CC . Common core structure of amyloid fibrils by synchrotron x‐ray diffraction. J Mol Biol. 1997;273:729–739. 9356260 10.1006/jmbi.1997.1348

[pro70296-bib-0063] Tittelmeier J , Sandhof CA , Ries HM , Druffel‐Augustin S , Mogk A , Bukau B , et al. The HSP110/HSP70 disaggregation system generates spreading‐competent toxic α‐synuclein species. EMBO J. 2020;39:e103954. 32449565 10.15252/embj.2019103954 PMC7327497

[pro70296-bib-0064] Waudby CA , Knowles TP , Devlin GL , Skepper JN , Ecroyd H , Carver JA , et al. The interaction of αB‐crystallin with mature α‐synuclein amyloid fibrils inhibits their elongation. Biophys J. 2010;98:843–851. 20197038 10.1016/j.bpj.2009.10.056 PMC2830463

[pro70296-bib-0065] Wentink AS , Nillegoda NB , Feufel J , Ubartaite G , Schneider CP , De Los Rios P , et al. Molecular dissection of amyloid disaggregation by human HSP70. Nature. 2020;587:483–488. 33177717 10.1038/s41586-020-2904-6

[pro70296-bib-0066] Wilhelm BG , Mandad S , Truckenbrodt S , Kröhnert K , Schäfer C , Rammner B , et al. Composition of isolated synaptic boutons reveals the amounts of vesicle trafficking proteins. Science. 2014;344:1023–1028. 24876496 10.1126/science.1252884

[pro70296-bib-0067] Wood SJ , Wypych J , Steavenson S , Louis JC , Citron M , Biere AL . Α‐Synuclein fibrillogenesis is nucleation‐dependent: implications for the pathogenesis of Parkinson's disease. J Biol Chem. 1999;274:19509–19512. 10391881 10.1074/jbc.274.28.19509

[pro70296-bib-0068] Xu S , Chen M , Feng T , Zhan L , Zhou L , Yu G . Use ggbreak to effectively utilize plotting space to deal with large datasets and outliers. Front Genet. 2021;12:774846. 34795698 10.3389/fgene.2021.774846 PMC8593043

[pro70296-bib-0069] Yoo JM , Lin Y , Heo Y , Lee YH . Polymorphism in alpha‐synuclein oligomers and its implications in toxicity under disease conditions. Front Mol Biosci. 2022;9:959425. 36032665 10.3389/fmolb.2022.959425 PMC9412080

