# Supplementary material for: Competing chaperone pathways in α‐synuclein disaggregation and aggregation dynamics
Source: Protein Sci. 2025 Sep 13;34(10):e70296. doi: 10.1002/pro.70296 (PMC12432404; doi:10.1002/pro.70296)
Supplement: Supplementary file 1 — Data S1. Supporting Information. [file PRO-34-e70296-s001.docx]

**Competing chaperone pathways in α-synuclein disaggregation and aggregation dynamics**

*Nicola K. Auld^1^, Shannon McMahon^1^, Nicholas R. Marzano^1^, Antoine M. van Oijen^1,2^, Heath Ecroyd*^1^*

^1^ Molecular Horizons and School of Science, University of Wollongong, Wollongong, NSW, Australia

^2^ Faculty of Medicine and Health, University of Sydney, Sydney, NSW, Australia

* Correspondence to Heath Ecroyd ([heathe@uow.edu.au](mailto:heathe@uow.edu.au)); Ph + 61 2 4221 3443

7. SUPPLEMENTARY MATERIAL

**
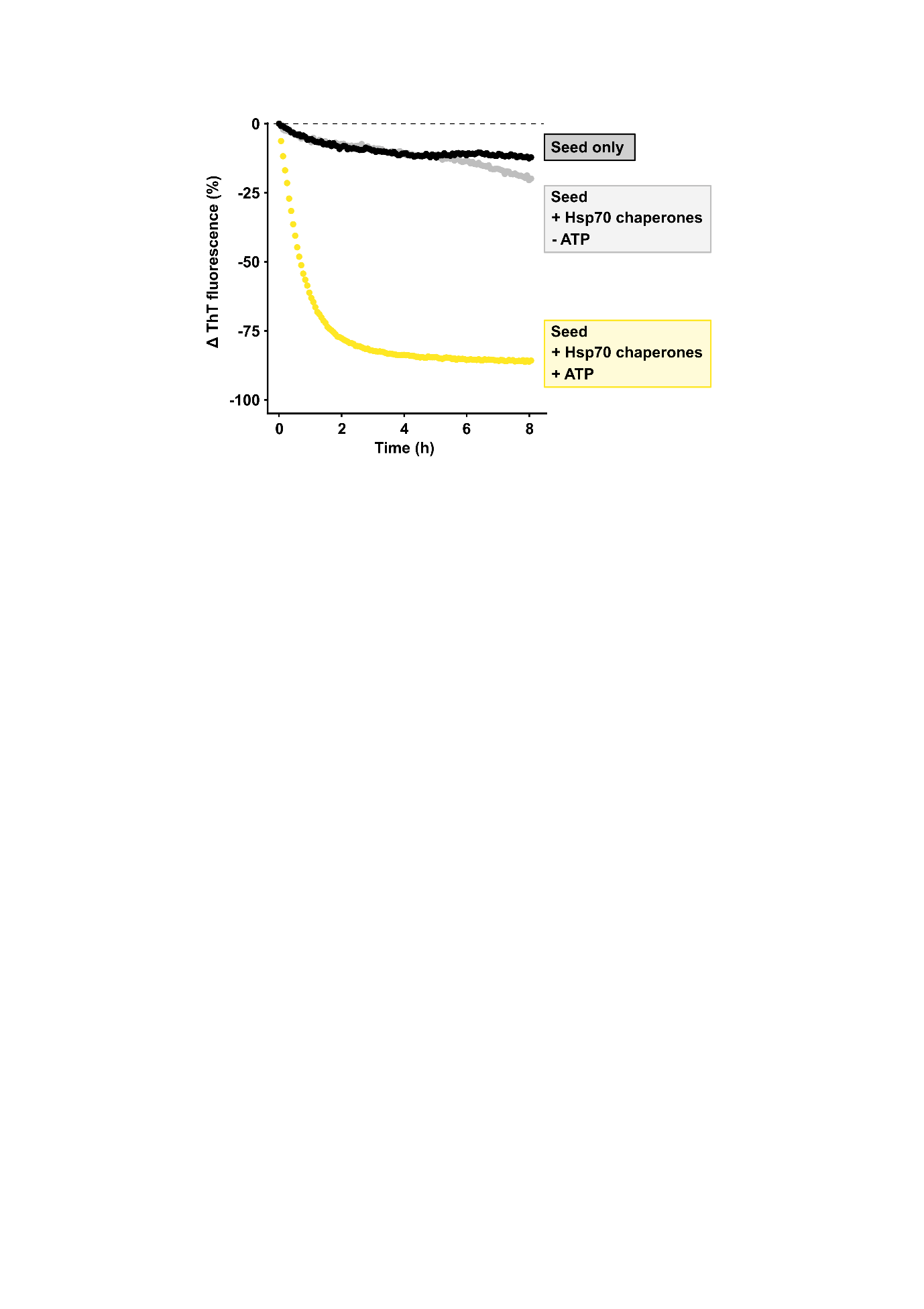
**

**Supplementary Figure 1. Hsp70-mediated disaggregation is ATP dependent.** α-synuclein seeds (2 µM) were incubated with Hsp70 chaperones (2 µM HspA8, 1 µM DNAJB1 and 0.2 µM Hsp110) in the presence or absence of ATP at 30^o^C for up to 8 h. Kinetic traces of α-synuclein seed and fibril disaggregation as monitored by the change in ThT fluorescence over time. Data are representative of three independent experiments.


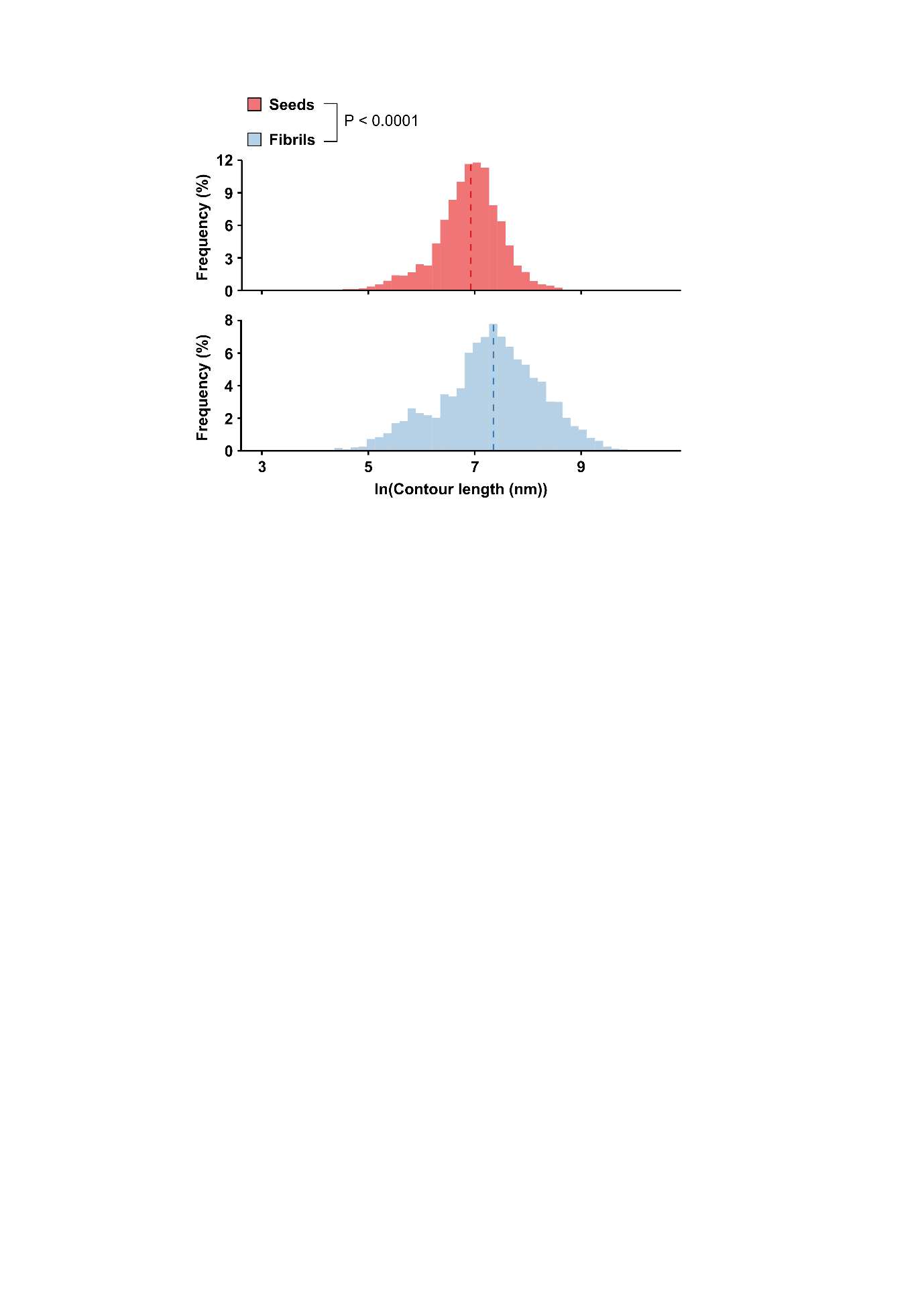


**Supplementary Figure 2. Sonication of mature α-synuclein fibrils produces shorter amyloid fragments (seeds).** Lengths of mature fibrils and α-synuclein seeds were calculated from TIRF microscopy images using the Ridge Detection plugin (Steger 1998) in ImageJ Fiji. Data were analysed using a two-tailed Welch’s t-test (n = 8609 identified contours for seeds and n = 3303 identified contours for fibrils taken from at least 30 images of each treatment from 2 independent experiments).


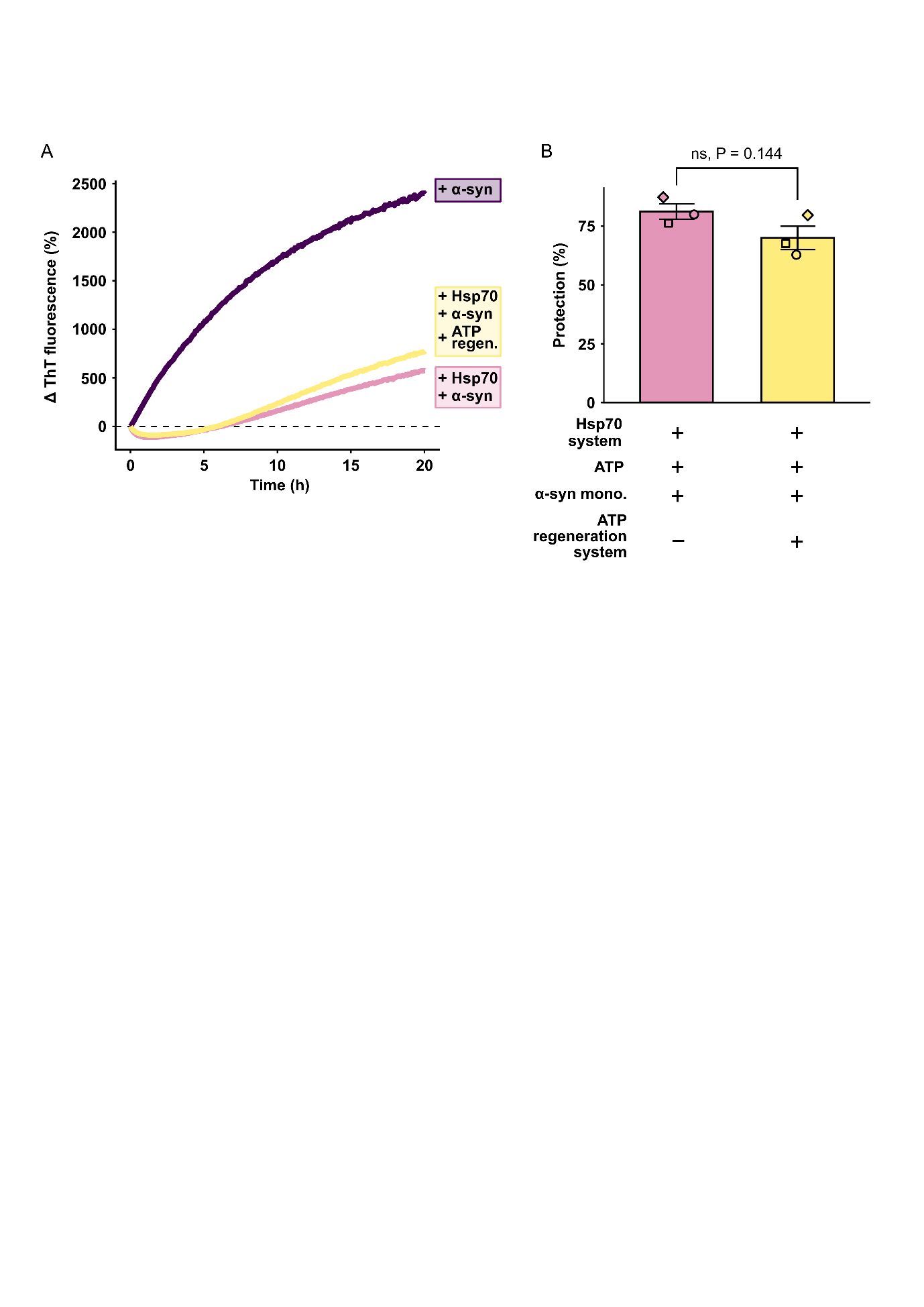


**Supplementary Figure 3. Hsp70 chaperones protect α-synuclein seeds against aggregation.** Physiologically relevant concentrations of Hsp70 system chaperones (14 µM HspA8, 7 µM DNAJB1 and 1.4 µM Hsp110) were incubated with α-synuclein seeds (2 µM), free monomeric α-synuclein (50 µM) and supplemented by an ATP regeneration system (8 mM PEP and 20 ng/µL pyruvate kinase). Representative traces are shown in **(A)**. The percentage protection against seed elongation afforded by Hsp70 chaperones with and without ATP regeneration system is shown in **(B)**. Data were analysed using a two-tailed Welch’s t-test (n = 3 independent experiments).
